# Highly flexible metabolism of the marine euglenozoan protist *Diplonema papillatum*

**DOI:** 10.1186/s12915-021-01186-y

**Published:** 2021-11-24

**Authors:** Ingrid Škodová-Sveráková, Kristína Záhonová, Valéria Juricová, Maksym Danchenko, Martin Moos, Peter Baráth, Galina Prokopchuk, Anzhelika Butenko, Veronika Lukáčová, Lenka Kohútová, Barbora Bučková, Aleš Horák, Drahomíra Faktorová, Anton Horváth, Petr Šimek, Julius Lukeš

**Affiliations:** 1 Institute of Parasitology, Biology Centre, Czech Academy of Sciences, České Budějovice (Budweis), Czech Republic; 2 Faculty of Natural Sciences, Comenius University, Bratislava, Slovakia; 3 Faculty of Science, Charles University, BIOCEV, Vestec, Czech Republic; 4 Faculty of Sciences, University of South Bohemia, České Budějovice (Budweis), Czech Republic; 5 Institute of Chemistry, Slovak Academy of Sciences, Bratislava, Slovakia; 6 Institute of Entomology, Biology Centre, Czech Academy of Sciences, České Budějovice (Budweis), Czech Republic; 7 Medirex Group Academy n.o., Trnava, Slovakia; 8 Faculty of Science, University of Ostrava, Ostrava, Czech Republic

**Keywords:** *Diplonema*, Metabolism, Multiomics, Hypoxia, Mitochondrion, Euglenozoa, Adaptation

## Abstract

**Background:**

The phylum Euglenozoa is a group of flagellated protists comprising the diplonemids, euglenids, symbiontids, and kinetoplastids. The diplonemids are highly abundant and speciose, and recent tools have rendered the best studied representative, *Diplonema papillatum*, genetically tractable. However, despite the high diversity of diplonemids, their lifestyles, ecological functions, and even primary energy source are mostly unknown.

**Results:**

We designed a metabolic map of *D. papillatum* cellular bioenergetic pathways based on the alterations of transcriptomic, proteomic, and metabolomic profiles obtained from cells grown under different conditions. Comparative analysis in the nutrient-rich and nutrient-poor media, as well as the absence and presence of oxygen, revealed its capacity for extensive metabolic reprogramming that occurs predominantly on the proteomic rather than the transcriptomic level. *D. papillatum* is equipped with fundamental metabolic routes such as glycolysis, gluconeogenesis, TCA cycle, pentose phosphate pathway, respiratory complexes, β-oxidation, and synthesis of fatty acids. Gluconeogenesis is uniquely dominant over glycolysis under all surveyed conditions, while the TCA cycle represents an eclectic combination of standard and unusual enzymes.

**Conclusions:**

The identification of conventional anaerobic enzymes reflects the ability of this protist to survive in low-oxygen environments. Furthermore, its metabolism quickly reacts to restricted carbon availability, suggesting a high metabolic flexibility of diplonemids, which is further reflected in cell morphology and motility, correlating well with their extreme ecological valence.

**Supplementary Information:**

The online version contains supplementary material available at 10.1186/s12915-021-01186-y.

## Background

The phylum Euglenozoa represents a group of flagellated protists known for major departures from that of a typical eukaryotic cell [1]. Euglenozoa consist of the predominantly parasitic kinetoplastids, free-living marine and freshwater diplonemids, and euglenids, as well as the poorly studied symbiontids [2, 3]. Diplonemids and kinetoplastids are united in the subphylum Glycomonada, based on the presence of the so-called glycosome, a peroxisome housing part of the glycolytic pathway [3].

Diplonemids were only recently shown to be one of the most speciose and abundant groups of marine eukaryotes of plankton communities [4–6]. Up to ~ 65,000 operational taxonomic units [7] are subdivided into four clades represented by just a handful of morphologically described species that are also available in culture [6, 8, 9]. The best studied representative, *Diplonema papillatum*, recently became amenable to genetic manipulations [10], enabling extensive future investigations in this model marine protist [11]. The nuclear genome of *D. papillatum*, however, remains in a preliminary assembly, mostly due to an abundance of highly repetitive content [12, 13]. Its mitochondrial genome encodes a standard set of genes [14], but multiplicated into the largest organellar genome known so far, in terms of total amount of DNA [15].

While parasitism is considered unlikely for most diplonemids [7, 16], we still do not know their lifestyle and primary energy sources. One can only surmise that these heterotrophic protists obtain essential compounds from the surrounding prokaryotic and/or eukaryotic plankton. Missing information about substrate preferences of *D. papillatum* precludes predictions of the metabolic pathways involved in meeting its energetic demands. The only metabolic study of this planktonic flagellate showed that it cannot take advantage of free glucose in the cultivation medium, but instead metabolizes amino acids [13]. Oxygen is a crucial parameter that determines how energy is obtained from substrates. As a constituent of zooplankton, *D. papillatum* relies on oxygen produced by phytoplankton during the day when sufficient light drives photosynthesis. However, during the night, respiration triggers a decrease in oxygen [17]. Since diplonemids have been reported from oxygen minimum zones [7], it is likely that they possess adaptations to fluctuating microaerobic concentrations of oxygen.

A general dearth of knowledge about the lifestyle of diplonemids forced us to apply a combined approach in order to map this protist’s metabolic complexity into a coherent framework. For that, we have performed differential gene expression analysis, and deep proteomic and metabolomic analyses of *D. papillatum*, grown under four different cultivation conditions. We mapped the recorded enzymes and metabolites across KEGG pathways and measured selected enzymatic activities. Analysis of the obtained data was aided by the extensive knowledge of the metabolism of related trypanosomatids and euglenids used here as reference points [2, 18]. Combined, these approaches allowed us to build the first robust model of diplonemid metabolism, which will improve our understanding of the ecological roles of this so far overlooked, yet indisputably important player in the oceanic environment.

## Results

### Morphology and growth under different conditions

**Fig. 1:**
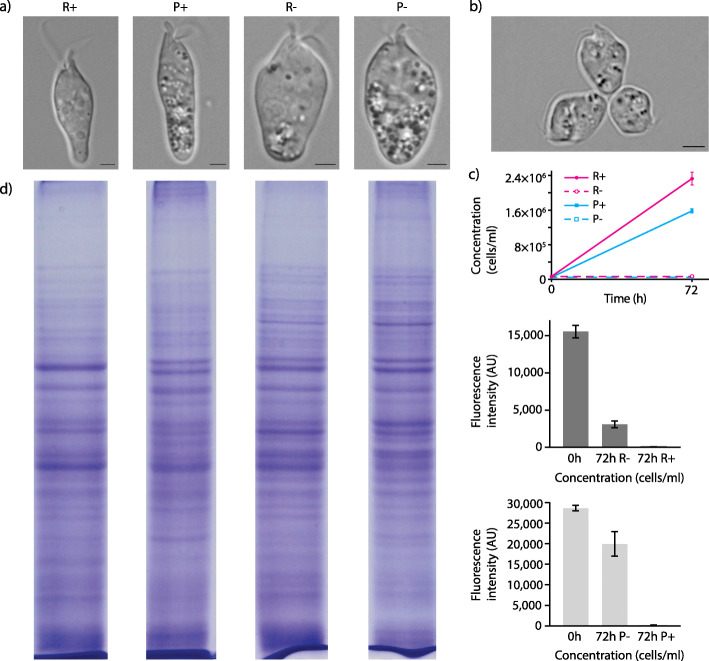
Morphology, growth, and protein profile of *D. papillatum* in different conditions. **a** Differential interference contrast images showing the altered morphology of *D. papillatum* cells grown under tested conditions. Scale bar 2.5 μm. **b** Rosettes formation. **c** Growth curves of *D. papillatum* and time-course changes in the mean fluorescence intensity emitted by cells under the tested conditions. Mean fluorescence intensity of cells maintained in rich medium significantly dropped while their concentration remained stable, thus indicating that cells underwent division but some also died. There was no significant difference in concentration of cells maintained in poor media; similarly, their mean fluorescence intensity dropped but insignificantly (*p* = 0.051), which is likely related to limited cell death. **d** Protein profiles of *D. papillatum* grown under tested conditions. Tested conditions: rich (R) and poor (P) media, aerobic (+) and hypoxic (-)

*D. papillatum* cells are usually elongated with a tapered posterior end, but under hypoxia, they acquire a rounded shape (Fig. 1a) and become markedly shorter (13.7 ± 2.2 μm and 17.5 ± 1.7 μm) than those in the oxygenated rich (17.1 ± 2.7 μm) and poor (22.6 ± 2.9 μm) media, respectively. Along with cell shortening, they increase in width from 5.1 ± 0.8 μm to 6 ± 1 μm in rich medium and from 5.5 ± 0.6 μm to 9.1 ± 1.8 μm in nutrient-depleted environment. In both aerobic and anaerobic conditions, cells cultured in poor medium invariably form numerous refractive vacuoles scattered throughout the cytoplasm (Fig. 1a). Being deprived of oxygen, the cells suspend progressive motion and start forming rosettes, in which they adhere to each other via their posterior ends (Fig. 1b).

Regardless of nutrient availability, under hypoxia, the population remained unchanged for 72 h (Fig. 1c). Still, cells kept in rich medium exhibited high proliferation, thus indicating their simultaneous decease. In poor medium, *D. papillatum* divided in significantly less (*p* = 0.051), but as reflected in their survival, the cells apparently tolerated hypoxia better (Fig. 1c).

### Omics of *Diplonema papillatum*

Different morphology observed in nutrient-rich medium with oxygen (R+) or in hypoxia (R-), and in nutrient-poor medium with oxygen (P+) or in hypoxia (P-) was associated with significant changes in the proteomic and metabolomic profiles (Fig. 1d; Additional file 1: Fig. S1; Additional file 2: File S1). Thus, we incorporated also transcriptomic analysis to gain a holistic view of changes in the metabolism of *D. papillatum*.

**Fig. 2:**
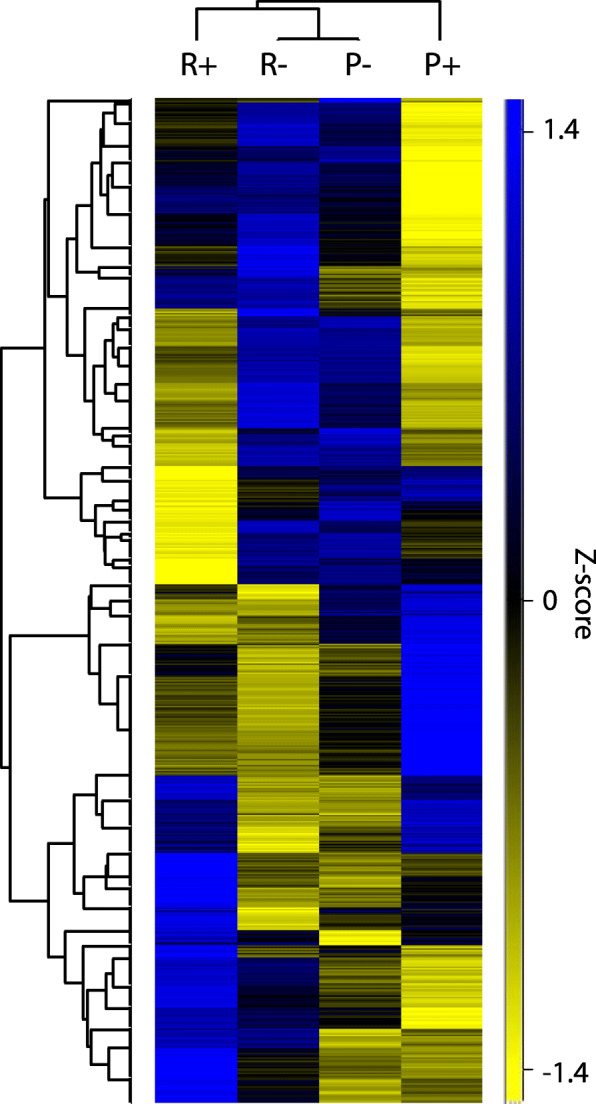
Heatmap of differentially abundant proteins identified in *D. papillatum*. *Z*-score-normalized averaged levels of proteins were clustered according to trend in particular experimental conditions: rich (R), poor (P), aerobic (+), and hypoxic (-). Data showed a dominant similarity based on the amount of nutrients in media (P versus R)

The transcriptome-derived protein dataset consists of 87,769 sequences. The relative levels of most transcripts were stable with only a few of them dysregulated under different cultivation conditions (Additional file 3: Table S1). However, the proteome showed significant changes in response to the cultivation conditions (Table 1; Additional file 3: Table S1). From 1900 proteins detected by mass spectrometry (MS), the levels of 913 were altered (Fig. 2). Altered protein levels discussed throughout the text represent log_2_-fold changes.

**Table 1:**
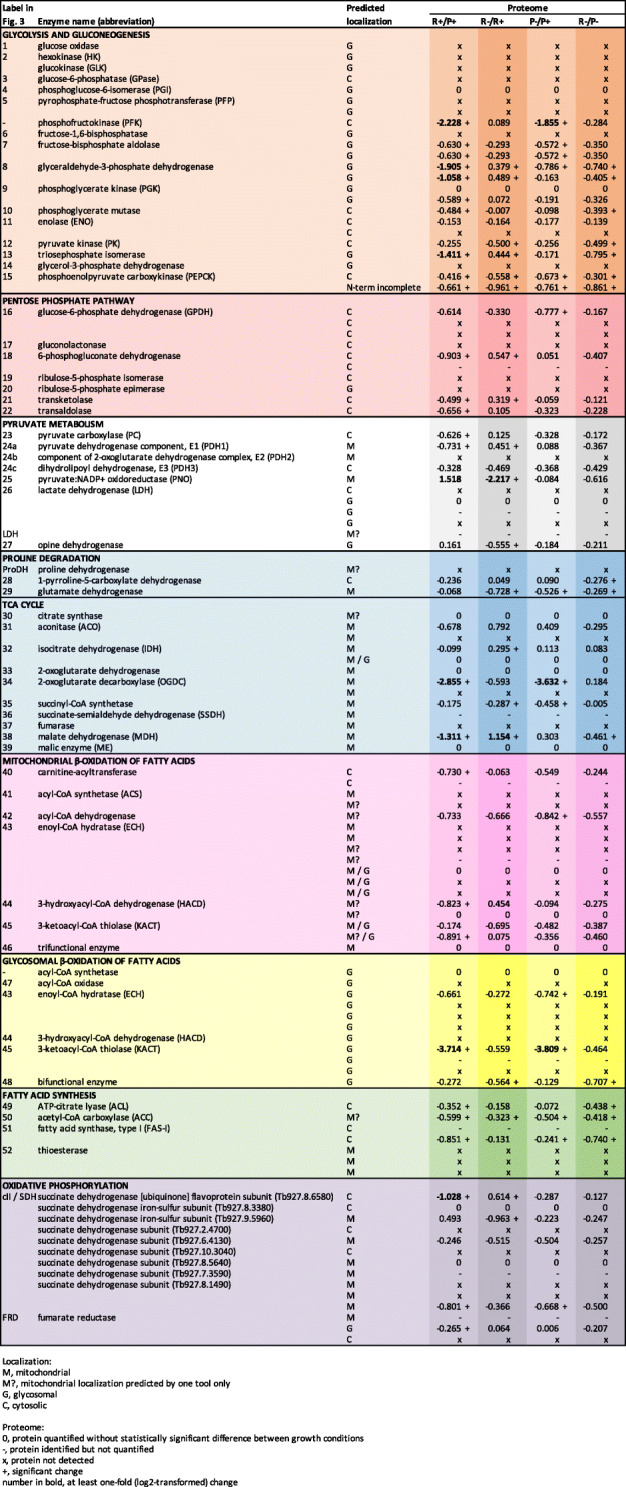
List of enzymes identified in transcriptome and proteome of *D. papillatum*. Abbreviations used for cellular localization and proteomic data are explained below the table. Transcriptomic and proteomic data are represented as log2-transformed ratios. ANOVA was performed with Benjamini-Hochberg correction for multiple testing with a *p*-value threshold at 0.01. For pairwise comparisons, post hoc Tukey’s test was used at *P* ≤ 0.01. Differentially abundant proteins were filtered on effect size, at least 1-fold of log_2_-transformed ratio. Abbreviations in column headings: rich medium (R), poor medium (P), aerobic conditions (+), hypoxic conditions (-)

Liquid chromatography-high-resolution mass spectrometry (LC-HRMS) used for mapping of the metabolome revealed the abundant presence of all major metabolites involved in central pathways, as well as major ionic species that shape osmolytic cell environment and represent important traits of the studied protist (Additional file 1: Fig. S1). For the list of 147 representative metabolites, see Additional file 4: Table S2. The metabolomic analysis of cells cultivated under different conditions showed their capability of rapid reprogramming, enabling robust adaptability to diverse and/or extreme environmental conditions.

In general, hypoxia caused neither upregulation of typical anaerobic enzymes, such as pyruvate:NADP^+^ oxidoreductase (PNO), fumarate reductase (FRD), and enoyl-coenzyme A (CoA) reductase, nor it had a marked effect on wax ester synthesis (Table 1; Additional file 3: Table S1). In any case, differences in nutrient availability had a major impact on protein levels (Fig. 2; Table 1; Additional file 3: Table S1). The transcriptome of *D. papillatum* contains enzymes ensuring activities of the following standard metabolic pathways: glycolysis, gluconeogenesis, tricarboxylic acid (TCA) cycle, pentose phosphate pathway (PPP), β-oxidation, and synthesis of fatty acids (FA), and the complete enzymatic set of oxidative phosphorylation (OXPHOS). Below, we characterize the main metabolic pathways and describe their alterations under different cultivation conditions. The outputs from transcriptomic and metabolomic analyses are combined in KEGG maps (Additional file 5: Data S1).

### Glycolysis and gluconeogenesis

**Fig. 3:**
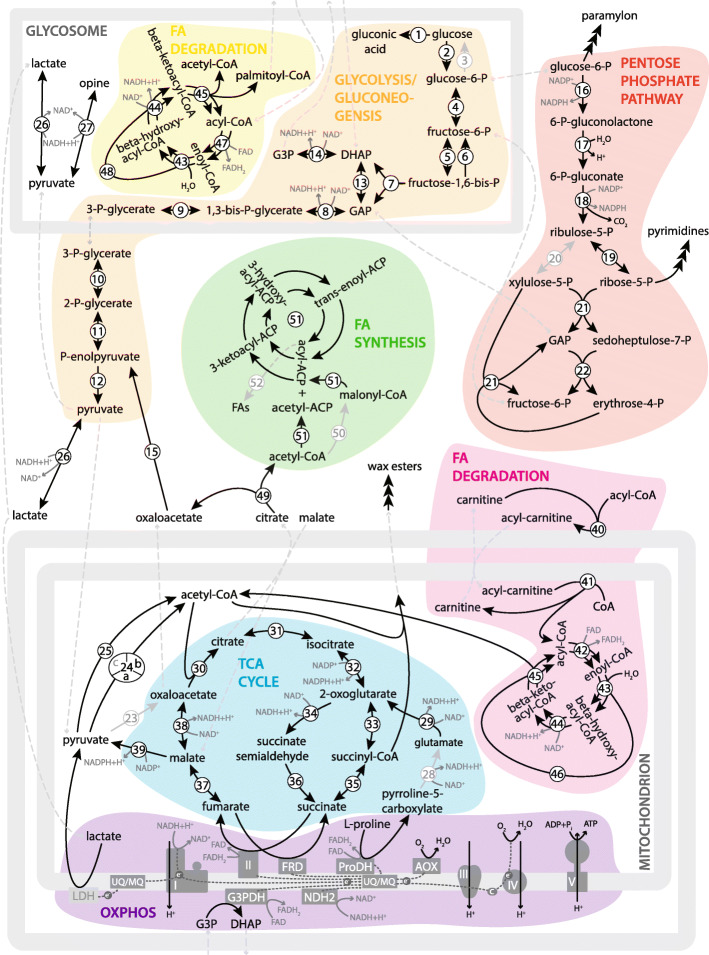
Metabolic pathways present in *D. papillatum*. Text and arrows in light-gray do not correspond to predicted localizations. Numbers correspond to enzyme numbers in Table 1: 1. glucose oxidase; 2. hexokinase (HK)/glucokinase (GLK); 3. glucose-6-phosphatase (GPase); 4. phosphoglucose-6-isomerase (PGI); 5. pyrophosphate fructose-6-phosphate 1- phosphotransferase (PFP); 6. fructose-1,6-bisphosphatase; 7. fructose-bisphosphate aldolase; 8. glyceraldehyde-3-phosphate dehydrogenase; 9. phosphoglycerate kinase (PGK); 10. phosphoglycerate mutase; 11. enolase (ENO); 12. pyruvate kinase (PK); 13. triosephosphate isomerase; 14. glycerol-3-phosphate dehydrogenase; 15. phosphoenolpyruvate carboxykinase (PEPCK); 16. glucose-6-phosphate dehydrogenase (GPDH); 17. gluconolactonase; 18. 6-phosphogluconate dehydrogenase; 19. ribulose-5-phosphate isomerase; 20. ribulose-5-phosphate epimerase; 21. transketolase, 22. transaldolase; 23. pyruvate carboxylase (PC); 24a. pyruvate dehydrogenase component, E1 (PDH1); 24b. component of 2-oxoglutarate dehydrogenase complex; E2 (PDH2), 24c. dihydrolipoyl dehydrogenase, E3 (PDH3); 25. pyruvate:NADP^+^ oxidoreductase (PNO); 26. lactate dehydrogenase (LDH); 27. opine dehydrogenase; 28. 1-pyrroline-5-carboxylate dehydrogenase; 29. glutamate dehydrogenase; 30. citrate synthase; 31. aconitase (ACO); 32. isocitrate dehydrogenase (IDH); 33. 2-oxoglutarate dehydrogenase; 34. 2-oxoglutarate decarboxylase (OGDC); 35. succinyl-CoA synthetase; 36. succinate-semialdehyde dehydrogenase (SSDH); 37. fumarase; 38. malate dehydrogenase (MDH); 39. malic enzyme (ME); 40. carnitine-acyltransferase; 41. acyl-CoA synthetase (ACS); 42. acyl-CoA dehydrogenase; 43. enoyl-CoA hydratase (ECH); 44. 3-hydroxyacyl-CoA dehydrogenase (HADC); 45. 3-ketoacyl-CoA thiolase (KACT); 46. trifunctional enzyme; 47. acyl-CoA oxidase; 48. bifunctional enzyme; 49. ATP-citrate lyase (ACL); 50. acetyl-CoA carboxylase (ACC); 51. fatty acid synthase, type I (FAS-I); 52. thioesterase. Abbreviations: AOX, alternative oxidase; CoA, coenzyme A; DHAP, dihydroxyacetone phosphate; FRD, fumarate reductase; G3P, glycerol-3-phosphate; G3PDH, glycerol-3-phosphate dehydrogenase; GAP, glyceraldehyde-3-phosphate; I-IV, respiratory complex I-IV; LDH, lactate dehydrogenase; MQ, menaquinone; NDH2, NADH dehydrogenase; ProDH, proline dehydrogenase; UQ, ubiquinone

The transcriptome of *D. papillatum* encodes all glycolytic enzymes, namely hexokinase (HK), glucokinase (GLK), phosphoglucose-6-isomerase (PGI), phosphofructokinase (PFK), aldolase, glyceraldehyde-3-phosphate dehydrogenase (GAPDH), phosphoglycerate kinase (PGK), phosphoglycerate mutase, enolase (ENO), pyruvate kinase (PK), and triosephosphate isomerase (Table 1). Moreover, all components of gluconeogenesis, such as glucose-6-phosphatase (GPase), fructose-1,6-bisphosphatase (FBPase), phosphoenolpyruvate carboxykinase (PEPCK), and pyruvate carboxylase (PC), were found as well (Table 1; Additional file 3: Table S1). The functionality of the above enzymes was corroborated by metabolomic analysis of their substrates (Additional file 4: Table S2). Based on the targeting sequences, steps 2 through 9 and step 13 of glycolysis occur in the glycosome, while steps 10 through 12 are located in the cytosol (Fig. 3; Table 1), which is in agreement with previous experimental and in silico analyses [13]. The same study identified two copies of PFK and claimed that glycosomal PFK1 is bacterial contamination, because in phylogenetic analyses it did not cluster with the eukaryotic PFKs, lacked a splice acceptor site, and no PFK activity was detected in the lysate [13]. However, due to our finding of PFK1 in the axenically cultured *D. papillatum*, contamination is highly unlikely, and we thus conclude that PFK1 was acquired horizontally from a bacterium. Moreover, previously found PFK1 [13] was truncated at its N-terminus (Additional file 1: Fig. S2) and, in fact, represents a pyrophosphate fructose-6-phosphate 1-phosphotransferase (PFP), which, being involved in both glycolysis and gluconeogenesis, catalyzes the forward and reverse reactions. We also identified a gene encoding the glycosomal PFP. Although transcripts for the above-described versions of PFK and PFP were identified, MS evidence is available only for the cytosolic version of PFK (Additional file 3: Table S1), which is clearly functional and is significantly upregulated under the P+ conditions when compared to both P- and R+.

**Fig. 4:**
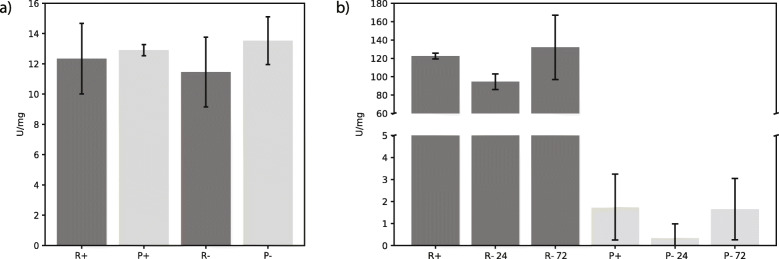
Hexokinase (HK) (**a**) and succinate dehydrogenase (SDH) (**b**) enzymatic activity. HK activity was measured in lysates and SDH activity was measured in mitochondrial lysates of cells cultivated in rich (R) and poor (P) media in aerobic (+) and hypoxic (-) conditions for 72 h only (HK) and for 24 and 72 h (SDH). Specific activity U was calculated as the amount of substrates converted by 1 μmol of enzyme per min. Single activities are represented by the average number from two biological replicates, each tested in three technical replicates, bars are standard deviations

Among the MS-identified proteins, we found neither high-affinity HK nor low-affinity GLK, yet specific activities corresponding to HK, GLK, or both were detected under all tested conditions (Fig. 4a). While the glycosome-targeted PFP and cytosolic GPase were present in the transcriptomes, they were not found in the analyzed proteomes (Table 1; Additional file 3: Table S1). The trace levels of ^14^C-glucose detected in lipids, FA, and monosaccharides (Additional file 1: Fig. S3A; Additional file 2: File S2) suggest a low level of glucose oxidation. Moreover, the metabolomic analysis detected several glycolytic substrates, including glucose and glucose-6-phosphate (Additional file 4: Table S2).

**Fig. 5:**
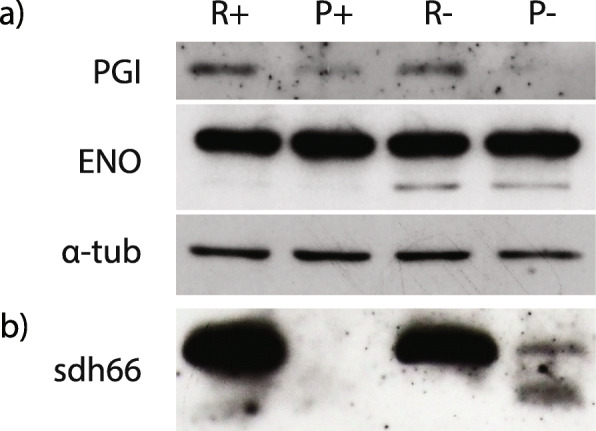
Western blot analysis of selected glycolytic (**a**) and TCA cycle (**b**) enzymes. Proteins were isolated from *D. papillatum* cells (**a**) or mitochondrial fractions from cells (**b**) cultivated in rich (R) and poor (P) media in aerobic (+) and hypoxic (-) conditions. α-tubulin was used as a loading control (A). ENO, enolase; PGI, phosphoglucose isomerase; α-tub; α-tubulin; sdh66, succinate dehydrogenase subunit I

Except for PGI, ENO, and PK, the glycolytic enzymes detected by the proteomic analysis were upregulated in poor medium (Table 1). Unexpectedly, MS revealed regulation of cytosolic but not glycosomal PGI (Additional file 3: Table S1). We showed by immunodetection that under all examined conditions PGI fluctuates, whereas the level of ENO remains stable (Fig. 5a; Additional file 2: File S3). Moreover, since the uptake of ^14^C-glucose was not increased by oxygen deprivation (Additional file 1: Fig. S3B and S3C) and the glucose levels remained stable (Additional file 4: Table S2), glycolysis is unlikely to be affected by the tested cultivation conditions. In contrast, the gluconeogenic activity seems to be modulated by altered conditions through the level of PEPCK that is positively stimulated by the presence of oxygen and nutrient deficiency (Table 1). Phosphoenolpyruvate, the metabolic product of PEPCK, is increased in R- over P- conditions (Additional file 4: Table S2). The absence of GPase from the proteomic data suggests that gluconeogenesis proceeds up to glucose-6-phosphate, which supplies the PPP (Additional file 4: Table S2).

### Pentose phosphate pathway

Metabolomic analysis revealed four circulating metabolites of the PPP (Additional file 4: Table S2) and very low levels of gluconate-phosphate. Sequence analysis of the PPP enzymes predicted their cytosolic localization in *D. papillatum*, except for ribulose-5-phosphate epimerase that possesses the glycosomal targeting signal (Table 1). However, the relocation of this sole PPP step into the glycosome is highly unlikely.

To investigate the regulation of PPP under tested conditions, we focused on the protein level of glucose-6-phosphate dehydrogenase (GPDH), which catalyzes its rate-limiting step and controls the ratio among glucose-6-phosphate, fructose-6-phosphate, and glyceraldehyde-3-phosphate, representing the entry and exit points for intermediate metabolites. An upregulation of GPDH and transaldolase, the last enzyme of PPP, occurred under P+ condition (Table 1).

**Fig. 6:**
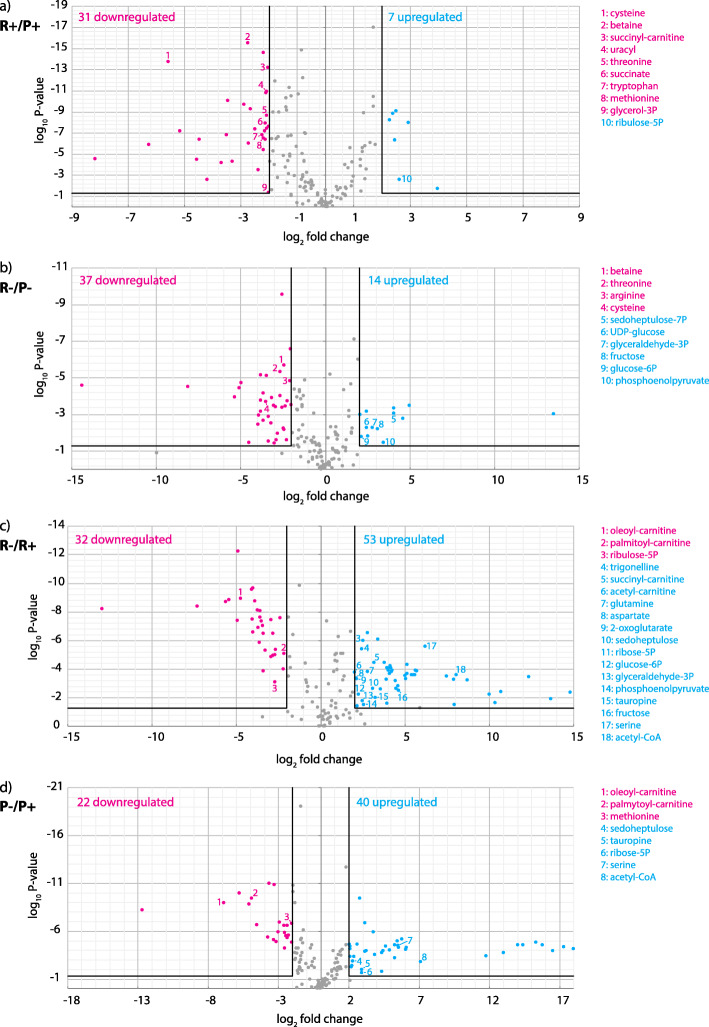
Relative quantitative analysis of selected metabolites under aerobic (+) and hypoxic (-) conditions. *D*. *papillatum* grown in rich (R) and poor (P) media were compared. The volcano plots of metabolite distribution show upregulated (blue) and downregulated metabolites (pink) compared in different conditions. Log_2_-fold change and log_10_ *p*-value significance cutoffs for differential expression were 2 and 0.05, respectively. Metabolites discussed in the text are numbered in plots and listed on the right, and others can be found in Additional file 4: Table S2

The ratio of metabolites between cells grown under R+ versus P+ conditions showed a decrease in the concentrations of glucose-6-phosphate, glyceraldehyde-3-phosphate, ribose-5-phosphate, ribulose-5-phosphate, and sedoheptulose-7-phosphate in poor medium. The differences were even more pronounced under hypoxic conditions (Fig. 6; Additional file 4: Table S2). The decrease of ribose-5-phosphate concentration can be related to growth retardation caused by the lack of nutrients (Fig. 1c) and the resulting low demand for pyrimidine nucleotides required for DNA synthesis during cell division. Downregulation of proteins involved in DNA replication and translation under the same condition also points at a slower cell division (Additional file 1: Fig. S4A and S4B). Elevated levels of GPDH, 6-phosphogluconate dehydrogenase, transketolase, and transaldolase under P+ conditions may reflect increased oxidative stress, and thus, the need for NADPH as an essential reductive coenzyme required for the activity of detoxifying systems such as glutathione reductase. Its protein level increased in poor medium (Additional file 1: Fig. S4C), although the relative level of input PPP metabolites was reduced (Fig. 6; Additional file 4: Table S2). Altogether, the levels of PPP enzymes and their metabolic substrates were substantially influenced by both nutrition and hypoxia, suggesting that *D. papillatum* may tolerate oxygen deprivation better than the stress caused by the lack of nutrients.

### Pyruvate metabolism

Pyruvate is a key intermediate generated via glycolysis and amino acid metabolism. For the reasons explained above, glycolysis (particularly the upper investment phase) is unlikely to be a major energetic source in *D. papillatum* under any of the tested conditions. We did not observe a significant change in pyruvate content between the P+ and R+ cells. However, the pyruvate turnover was enhanced under P-, while phosphoenolpyruvate decreased 3.4-fold (Fig. 6; Additional file 4: Table S2). It suggests that the rate of glycolysis still depends on the cultivation conditions, yet the origin of glucose entering glycolysis remains uncertain. Although *D. papillatum* possesses a sodium/glucose cotransporter (Additional file 3: Table S1), only an insignificant amount of ^14^C-glucose was taken up from the medium where glucose was the only available energy source, even upon starvation for 12 h in sea salt solution (Additional file 1: Fig. S3B and S3C).

Nevertheless, poor nutritional conditions triggered a significant production of glucogenic amino acids that can be deaminated to pyruvate, in particular threonine, glycine, cysteine, aspartate, and tryptophan (Fig. 6; Additional file 4: Table S2), which indicates pyruvate biosynthesis via the transamination reactions. This catabolic process is further enhanced in the hypoxic cells (particularly in the case of serine and aspartate) and corresponds well with the upregulation of pyruvate in P- condition. The data on pyruvate transaminases further corroborate this observation and selective action of participating enzymes under different stress conditions. For instance, the relative abundance of aspartate transaminase was increased in hypoxia in both types of media, while the level of alanine transaminase remained unchanged (Additional file 3: Table S1), corresponding well to the acquired metabolomic data (Additional file 4: Table S2).

*D. papillatum* is equipped with several biochemical pathways for the metabolism of pyruvate. Five distinct sequences of lactate dehydrogenase (LDH) have been detected, one cytosolic, three targeted to the glycosome, and one possibly to the mitochondrion (Table 1). The glycosomal and cytosolic forms mediate the conversion of pyruvate to lactate by producing NAD^+^ during anaerobic metabolism, while the mitochondrial LDH catalyzes the oxidation of lactate in the organellar matrix without expending NADH. Under all studied cultivation conditions, the LDH level remained unchanged (Table 1) and the fluctuation of lactate levels in the metabolomic data was statistically insignificant (Additional file 4: Table S2).

We found three pyruvate reductive condensation products with particular amino acids that are historically called opines [19], namely those with 3-alanine, taurine, and lysine known as 3-alanopine, tauropine, and saccharopine, respectively (Additional file 4: Table S2). We identified only a single, amino acid-nonspecific opine dehydrogenase (OPDH), which was not increased in hypoxia, while the protein level was upregulated in both oxygenated poor and rich media (Table 1). The low nutrition increases the opine levels, and tauropine and saccharopine further rise in hypoxia, while 3-alanopine dropped in P- conditions (Additional file 4: Table S2). This is consistent with a lower relative abundance of 3-alanine in cells cultured under these conditions (Additional file 4: Table S2).

To investigate the origin of *D. papillatum* OPDH and its homologue in related *Euglena gracilis*, we conducted a phylogenetic analysis. The OPDH dataset of the mollusk/annelid type and the sponge type that belongs to the ornithine cyclodeaminase/mu-crystallin family studied previously [19] was expanded by incorporating more eukaryotic and prokaryotic sequences. The maximum likelihood phylogenetic tree revealed a clear affiliation of the euglenozoan sequences with the mollusk/annelid type (Additional file 1: Fig. S5), suggesting that these protists acquired their OPDH via horizontal gene transfer (HGT) from a bacterial source.

The OPDH of *D. papillatum* unexpectedly possesses the glycosomal targeting signal, whereas pyruvate kinase, which converts phosphoenolpyruvate to pyruvate, is predicted to be cytosolic (Table 1). Consequently, one would expect that the conversion from pyruvate to opine occurs in the cytosol. However, the glycosomal targeting signals in three LDHs and OPDH strongly indicate either pyruvate import to the glycosomes or pyruvate formation inside the organelle. The transaminases identified in the proteome, namely aspartate and possibly 4-aminobutyrate, are located in the mitochondrion, while according to the prediction programs, alanine transaminase is either mitochondrial or glycosomal (Additional file 3: Table S1). Thus, pyruvate production via transamination inside the glycosome might be feasible. This observation indicates a unique organellar compartmentalization and the capability of permanent simultaneous aerobic/anaerobic glycolytic energy production.

Pyruvate NADP^+^ oxidoreductase (PNO) is an enzyme characteristic for hydrogenosomes that functionally replaces the activity of pyruvate dehydrogenase complex (PDH) in these organelles. Since neither the transcript nor the protein level of PNO was influenced by oxygen concentrations (Table 1), it is plausible that under the tested conditions, the activities of PNO and PDH are interchangeable. However, despite the presence of several putative subunits of PDH, we do not know whether it is functional.

### TCA cycle

While the complement of the glycolytic enzymes is conserved across the euglenozoans, their TCA cycle, along with oxidative phosphorylation and β-oxidation of FAs, varies in both the enzymatic composition and mode of operation. *D. papillatum* bears standard TCA cycle enzymes but also 2-oxoglutarate decarboxylase (OGDC) and succinate-semialdehyde dehydrogenase (SSDH) (Table 1). Several of the corresponding genes are multicopy, with at least one version of their protein product targeted into the mitochondrion (Table 1).

In *D. papillatum*, the amino acids represent a significant carbon source for TCA (see above), and their reduced supply induces changes in morphology and protein profile (Fig. 1a, d). Proline is another key intermediate presumably feeding the TCA cycle via proline dehydrogenase (ProDH). Multiple functions of proline are reflected by the identification in the transcriptome of several enzymes involved in proline degradation, namely ProDH, pyrroline-5-carboxylate dehydrogenase, glutamate dehydrogenase, and pyrroline-5-carboxylate reductase, although the proteomic evidence for the first enzyme is missing (Table 1, Additional file 3: Table S1).

Out of three fumarate reductases (FRDs) recognized in the transcriptome, one is predicted to have mitochondrial, one cytosolic, and one glycosomal localizations (Additional file 3: Table S1). Their transcript and protein levels were not significantly affected by oxygen, but the glycosomal version was more abundant in P+ compared to R+ conditions (Table 1, Additional file 3: Table S1). While in *D. papillatum* transcripts are generally not affected by different growth conditions, protein levels of some TCA enzymes were significantly altered by cultivation conditions (Table 1). Normoxia led to the accumulation of the only mitochondrial succinyl-CoA synthetase and OGDC, whereas mitochondrial ACO and mitochondrial malate dehydrogenase (MDH) were upregulated under low-oxygen concentration in both types of media. MDH was found in three copies with predicted mitochondrial, glycosomal, and cytosolic localizations. In both media, the dynamics of glycosomal MDH is opposite to its mitochondrial and cytosolic homologues (Additional file 3: Table S1), suggesting that under hypoxia, malate conversion in the mitochondrion and/or cytosol dominates over the glycosomal one. Under these conditions, glycosomal malate may arise from the conversion of phosphoenolpyruvate to oxalacetate by PEPCK with concomitant fixation of CO_2_ and ATP synthesis, and further activity of glycosomal MDH. In the mitochondrion, malate arises from pyruvate conversion via malic enzyme (ME), and the metabolite is then exported to the cytosol (Fig. 3). Such transport is predicted based on the absence of fumarase from the proteomic data, although due to the detection of a transcript corresponding to mitochondrion-targeted fumarase and the presence of fumarate in the metabolomic data (Additional file 4: Table S2), *D. papillatum* seems to have the capacity to produce fumarate (Table 1). A significant increase in the relative protein level of cytosolic and mitochondrial MDH under hypoxic conditions manifested their functional importance regardless of the available nutrients. Citrate/isocitrate, 2-oxoglutarate, and succinyl-CoA were more abundant under hypoxia in rich medium as compared to poor medium, while the opposite trend was observed for succinate, fumarate, and malate (Fig. 6; Additional file 4: Table S2).

From five isocitrate dehydrogenases (IDHs), two each have mitochondrial, glycosomal, and one cytosolic localization. In the proteome only, the mitochondrial and glycosomal versions were detected (Additional file 3: Table S1). As a rate-limiting step of TCA, a consistent presence of IDH may reflect the stable flow of the TCA cycle despite fluctuations in nutrients. However, variations in the specific activity of succinate dehydrogenase (SDH/complex II) and in the amount of flavine subunit manifest an existing regulation. Based on sequence homology with the related *Trypanosoma brucei*, we identified nine SDH subunits (one in three copies), three of which lack the mitochondrial import signal (Table 1). There is proteomic evidence for five subunits uninfluenced by the presence or absence of oxygen in the poor medium, while under aerobic conditions and high nutrient content, at least four SDH subunits accumulated. Based on the MS data, the flavine subunit was more abundant in poor medium under normoxia (Table 1), although both the activity measurement and immunodetection indicated a reduction of the SDH complex under this condition. We analyzed the specific activity after 24 and 72 h of hypoxia in both types of medium. In poor and rich media, the SDH activity was 0 and ~ 132 ± 35 U/mg, respectively (Fig. 4b). The absence of activity in both P+ and P- cells corresponds well with the lack of a signal in the former and a weak signal in the latter, as shown by western blotting using antibodies recognizing flavine subunit (Fig. 5b; Additional file 2: File S3). However, in the MS data, the protein accumulated in P+ as compared to R+ condition (Table 1). Such discrepancy may be explained by post-translational modifications, which were outside of the scope of this work.

The metabolomic data, which reflect the total flow of substrates through the TCA cycle, provided two different relative abundance patterns, one for malate-fumarate and another for the citrate-isocitrate-aconitate segment (Additional file 4: Table S2). They confirmed the prevailing perturbation of malate and fumarate under hypoxia, a rather uniform pattern of the relative citrate-isocitrate-aconitate abundance and different fluctuations of succinate and 2-oxoglutarate in the studied cells (Additional file 4: Table S2).

The incorporation of ^14^C-proline-derived carbon into the lipids and FA (Additional file 1: Fig. S3A; Additional file 2: File S2) is compatible with the reductive way of the TCA cycle, operating from 2-oxoglutarate to citrate, which is transported through the malate exchange into the cytosol. Citrate is subsequently split into oxaloacetate and acetyl-CoA via the cytosolic ATP-citrate lyase (Fig. 3), which is significantly upregulated in the poor as compared to the rich medium regardless of the presence of oxygen (Table 1). Oxaloacetate can be converted to phosphoenolpyruvate via PEPCK, as described above, and acetyl-CoA enters the FA synthesis (see below).

### Oxidative phosphorylation

While we found homologues of numerous subunits of respiratory complexes II through V (Additional file 3: Table S1), the likely identification of a high number of divergent and/or species-specific subunits will be possible only following the isolation and purification of these complexes. Along with ProDH and the mitochondrial LDH, alternative NADH dehydrogenase, alternative oxidase, glycerol-3-phosphate dehydrogenase, and enzymes for the menaquinone and ubiquinone syntheses (Additional file 3: Table S1) combined demonstrate a highly flexible structure and composition of OXPHOS in *D. papillatum*, allowing efficient respiration and ATP synthesis from a range of substrates.

### β-oxidation of fatty acids

*D. papillatum* produces a wide range of FA. Metabolomic analysis confirmed the variable abundance of a set ranging from propionate (C3:0) to docosapentaenoate (C22:5n3) (Additional file 4: Table S2). The FA oxidation takes place in both the mitochondria and glycosomes. Before oxidation, due to the inability of acyl-CoA to cross the mitochondrial membrane, the acyl group is transferred from the cytosol to the organelle in the form of acyl-carnitine. Carnitine-acyltransferase, which catalyzes the rate-limiting step in β-oxidation, namely the transfer of acyl to carnitine, is encoded by two highly similar transcripts with predicted cytosolic localization, and the proteomic data for the product of one of them showed its level upregulated in poor as compared to rich medium. We observed a significant increase of free carnitine, its lower acyl forms, acetyl-CoA, and propionyl-CoA, particularly under hypoxic conditions (Additional file 4: Table S2), which suggests rapid recycling of acyl-carnitine into carnitine and acyl-CoA. On the other hand, metabolism of carnitines and FA with longer acyls seems to be suppressed under hypoxia (Additional file 4: Table S2). FA enter the oxidation in the form of acyl-CoA that is synthesized by acyl-CoA synthetase (ACS) residing in the inner mitochondrial membrane (Fig. 3). We identified seven transcripts corresponding to this enzyme, with the proteomic evidence available for only two of the cytosolic versions of ACS (Additional file 3: Table S1).

The first step of β-oxidation is performed by acyl-CoA dehydrogenase in the mitochondria and acyl-CoA oxidase in the glycosomes, with the remaining three steps catalyzed by the same enzymes, namely enoyl-CoA hydratase (ECH), 3-hydroxyacyl-CoA dehydrogenase (HACD), and 3-ketoacyl-CoA thiolase (KACT). While the glycosomal bifunctional enzyme performs the second and third steps, the mitochondrial trifunctional enzyme combines activities of the second, third, and fourth steps. The detected product of both pathways is a FA shortened by one acetyl-CoA (Fig. 3; Table 1). Most enzymes are encoded by multiple transcripts, with at least one copy being targeted to the corresponding organelle (Additional file 3: Table S1). One copy each of the mitochondrial and glycosomal ECH, HACD, and KACT was upregulated in P+, with the peptides for the other copies absent from the proteomic data. The accumulation of FAs was generally higher in P+ than in R+, and it is also corroborated by elevated fluxes of higher FAs in P+ condition (Additional file 4: Table S2).

### Fatty acid and wax ester syntheses

Eukaryotes employ two pathways for the FA synthesis, namely the cytosolic multidomain type I FA synthase (FAS-I) and the mitochondrial and plastidial type II FA synthase (FAS-II), with each step catalyzed by independent enzymes. We identified only FAS-I (Fig. 3; Table 1) that seems to operate in the cytosol. The MS data contained two proteins corresponding to FAS-I. One version exhibited stable levels under all tested conditions, while there were changes in the levels of the other FAS-I version, and ATP-citrate lyase (ACL) and acetyl-CoA carboxylase (ACC) that precede FAS-I. Both ACL and ACC were similarly upregulated in normoxia, being more abundant in P+ than in R+ conditions (Table 1). Elongation of FAs via the set of elongases is possible in *D. papillatum*; however, from nine transcripts, the only one identified in the proteome was significantly upregulated in poor medium (Additional file 3: Table S1).

Based on the sequence homology with *E. gracilis*, in the transcriptome of *D. papillatum*, we found all enzymes required for wax ester synthesis, furnished with the mitochondrial import signal. The first four enzymes of the pathway, namely methylmalonyl-CoA mutase, propionyl-CoA carboxylase, 3-ketoacyl-CoA thiolase, and 3-hydroxyacyl-CoA dehydrogenase, were detected by MS with significant regulation (Additional file 3: Table S1).

### Unusual metabolites

Our metabolome analysis revealed several unusual metabolites (Additional file 4: Table S2), with their novelty being considered based on their general occurrence, structural uniqueness, or both (Additional file 6: Data S1). The identification of highly abundant betaine (glycerol methyl-3-alanine betaine) and a minor isomeric glycerol-N-trimethyl homoserine (GTS) that along with glycerophosphocholine belong to osmolytes due to their colligative properties turned our attention to a plausible occurrence of their lipid species mono- (MGTA) and diacyl glycerol-3-trimethyl alanine (DGTA), called betaine lipids. We identified a comprehensive set of 3-alanine-derived MGTA and DGTA lipids and compared them with the analogously functioning phosphatidylcholines (Additional file 4: Table S2). The MGTA species provide complex but not unambiguously interpretable abundance patterns (Additional file 4: Table S2). Some metabolites, such as MGTA-C18:3, are most abundant in R+ normoxia, while others, such as MGTA-20:2-5n, dominate in P- conditions (Additional file 4: Table S2). The detected trigonelline, gonyol, and 3-methylsulfoniopropionate may function as unusual potential osmolytes in a non-photosynthetic organism (Additional file 4: Table S2).

## Discussion

Diplonemids belong among the most abundant marine planktonic protists [20]. While their ecological functions remain unknown, they take up nutrients most likely via the cytostome [21], may prey on bacteria [22], and have also been associated with parasitism [16]. In this work, the model species *D. papillatum* was subjected to the analysis of the fundamental energetic pathways compartmentalized in its cytosol, mitochondrion, and specialized peroxisomes, known as glycosomes [3]. Based on the KEGG-mapped metabolism, we predicted the most plausible energetic sources, which these protists may use in the wide ecological niche they occupy. As anticipated from their presence in oxygen minimum zones [7], due to the presence of PNO, FRD, enoyl-CoA reductase, and wax ester synthesis enzymes, diplonemids have the potential to adapt to a low-oxygen environment. Hypoxia is a strong positive regulator of the expression of anaerobic enzymes in many organisms [23–25]. However, in *D. papillatum*, such regulation was not observed, suggesting a constitutive expression of these enzymes, enabling the cells to efficiently cope with alterations of the aerobic and hypoxic environments.

*D. papillatum* synthesizes ubiquinone and menaquinone, the latter known to be involved in fumarate reduction via FRD in prokaryotes [26]. The related *E. gracilis* employs FRD in hypoxia but in the same pathway uses rhodoquinone instead of menaquinone [27]. Upregulation of ubiquinone/menaquinone synthesis in the absence of oxygen may reflect the demand for electron transporter, which may stimulate reduced respiration. Our results suggest that the dissected diplonemid constitutively produces enzymes and cofactors required under hypoxia, including FRD and menaquinone [28], which are then readily available when the flagellate enters anaerobic conditions. In a somewhat less flexible setup, the related euglenids maintain low levels of anaerobic enzymes under aerobic conditions [29, 30] and, upon oxygen deprivation, upregulate PNO and enoyl-CoA reductase [30].

The situation is different in the parasitic *T. brucei*, which harbors FRD and can survive anaerobiosis only for a short period of time, excreting succinate into the medium [31]. Succinate arises in the glycosome, where it is produced from imported phosphoenolpyruvate by PEPCK, MDH, fumarase, and FRD [32]. In the free-living euglenids, succinate undergoes conversion to propionyl-CoA, which then enters wax ester synthesis, a unique energy-gaining pathway enabling them to survive anaerobiosis [33]. In *D. papillatum*, MDH and FRD carry the glycosomal targeting signal, but its absence in PEPCK, along with the only identified fumarase equipped with the mitochondrion targeting signal (Table 1), makes the production of succinate in the glycosomes highly unlikely. A high accumulation of succinate in poor medium under aerobic conditions correlates with the absence of fumarase from the proteomic data. However, at present, we cannot rule out the possibility that malate is supplied by the conversion of pyruvate from amino acids and that the detected fumarate is a product of an alternative reaction from phenylalanine and tyrosine, as seen in the parasitic kinetoplastid *Leishmania* [34].

In the TCA cycle of anaerobic eukaryotes, FRD and SDH catalyze opposite reactions, with the expression of the latter key enzyme that connects TCA and OXPHOS regulated by the concentration of oxygen [25]. In *D. papillatum*, it is the availability of nutrients, not oxygen, that seems to be critical for the SDH activity. Its dramatic decrease inevitably disrupts the TCA cycle as a response to a limited carbon source. Following the accumulation of succinate in poor medium under aerobic conditions, the activity of SDH became undetectable in the mitochondrion, regardless of the abundance of this protein in cell lysates. Alternatively, FRD may replace SDH in either oxidative or reductive way of the TCA cycle, as was shown in some prokaryotes [35].

Although the TCA cycle is considered a universal metabolic pathway, some euglenozoans evolved significant departures in this respect, exemplified by the procyclic stage of *T. brucei* that has repurposed parts of its TCA cycle and does not use it for energy generation [36, 37]. When operational, TCA cycle in *T. brucei* is not fueled by acetyl-CoA from glycolytic pyruvate but by amino acids, mainly proline and threonine, because glucose is converted to glycosomal succinate, alanine, and acetate rather than pyruvate [38, 39]. Since *E. gracilis* lacks two typical TCA cycle enzymes, namely 2-oxoglutarate dehydrogenase and succinyl-CoA synthetase, it is bound to utilize an alternative route, in which OGDC, essential for the aerobic growth, catalyzes the conversion of 2-oxoglutarate to succinate semialdehyde, in turn oxidized by SSDH to succinate [24, 40]. This situation is not unprecedented, since cyanobacteria also possess a modified TCA cycle employing OGDC and SSDH [41]. However, the co-existence of modified and classical TCA cycle enzymes as described here for *D. papillatum* is unique, underlying once again its characteristic metabolic versatility (Fig. 3). Our data suggest the physiological involvement of both pathways under certain conditions. While the studied protist maintains a stable level of 2-oxoglutarate dehydrogenase, its physiological counterpart OGDC is significantly affected by nutrient availability, as well as by oxygen. The regulation at protein level signifies an alternative route being active in the poor medium under aerobic conditions, while in the rich medium and oxygen deprivation, the standard route via 2-oxoglutarate dehydrogenase and succinyl-CoA synthetase dominates. Functional analysis of individual enzymes will shed light on the physiological connection of these two alternative pathways. Our data indicate that under exogenous stress, when the electron transport chain operates weakly and high-energy phosphates are generated mainly through matrix substrate-level phosphorylation, the TCA cycle pathway remains operational, yet may be segmented, exhibit opposite directionalities, and/or may be fed by amino acids.

Although fully equipped with the aerobic enzymes, the facultatively anaerobic mitochondrion of *E. gracilis* utilizes PNO, a hallmark enzyme of hydrogenosomes [42], for pyruvate oxidation under anaerobiosis [43]. So far, *E. gracilis* was unique by harboring PNO in its fully functional mitochondrion [44]. PNO is thought to play a pivotal role in wax ester synthesis, which occurs when cells face the low-oxygen conditions, while in the presence of oxygen, they compensate for the decrease of PNO by increasing PDH, which readily supplies the TCA cycle with acetyl-CoA. Both enzymes can co-exist, as was shown in *E. gracilis*, in the aerobic mitochondrion of which PNO retains its activity, indicating that the enzyme is not as oxygen-labile in vivo as it is in vitro [24]. While in *D. papillatum*, fluctuations of the PNO protein levels do not correlate with oxygen, a regulation of the downstream pathways, the β-oxidation of FA and wax ester synthesis, occurred. Indeed, the absence of oxygen increased the level of propionyl-CoA carboxylase, an enzyme of the wax ester synthesis. At the same time, enzymes that initiate β-oxidation, carnitine-acyltransferase and acyl-CoA synthetase, were less abundant. This suggests that it is not PNO but components of the downstream pathways that determine whether β-oxidation of FA or wax ester synthesis, running in the opposite direction, prevails.

Due to the transcriptomic evidence for all enzymes of the proline degradation pathway, the most plausible scenario is that proline is degraded to 2-oxoglutarate, which then enters the TCA cycle. None of the enzymes involved in proline degradation responded to nutrient or oxygen fluctuation, except for glutamate dehydrogenase, the mitochondrial form of which was significantly upregulated in the presence of oxygen, regardless of the growth conditions. Once the proline-derived carbon enters the TCA cycle, in both oxidative and reductive directions, it can be metabolized to form citrate, the precursor of FA synthesis. While reductive carboxylation takes place in hypoxia [24], citrate synthesis in oxidative direction seems to be more likely, as succinyl-CoA synthetase was more abundant under the aerobic conditions. Counterintuitively, all TCA metabolites except for citrate accumulated in cells cultured in the poor medium. Citrate may either re-enter the TCA cycle and undergo conversion via ACO or can be exchanged for malate and metabolized in the cytosol to oxaloacetate and acetyl-CoA via ACL, which was more abundant under these conditions. Moreover, the upregulation of the two downstream enzymes (ACC and PEPCK) suggests that in the oxygenized poor medium, citrate is decomposed in the cytosol rather than oxidized in the TCA cycle. In *D. papillatum*, acetyl-CoA derived from citrate potentially enters the FA synthesis via cytosolic FAS-I, a pathway shared with *E. gracilis* (Fig. 3). This protist contains two pathways of the FA synthesis, namely FAS-I in the cytosol and FAS-II in the plastid and mitochondrion [43, 45]. This again suggests that diplonemids and euglenids resemble the last euglenozoan common ancestor more than the highly diverged kinetoplastids. In *D. papillatum*, the protein level of one copy of FAS-I remains unaltered by nutrients or oxygen, but the level of the second copy changes under the tested cultivation conditions. The level of ACL and ACC hinted at their involvement in the regulation of FA synthesis. Indeed, a pivotal role of ACC in this pathway is conserved from bacteria to humans [46], with the studied flagellate being no exception.

The accumulation of PC and PEPCK in cells grown in oxygenated poor medium supports the incorporation of oxaloacetate into gluconeogenesis, the intermediates of which are transformed into glucose. Alternatively, they are employed in the PPP, which is generally a major source of the NADPH equivalents required for the activity of enzymes involved in oxidative stress protection and for FA and pyrimidine nucleotide syntheses [47]. Although the PPP can completely convert glucose-6-phosphate to CO_2_, it also supplies glyceraldehyde-3-phosphate and fructose-6-phosphate to glycolysis. Depending on cellular requirements, the pathway can thus operate as a cycle [47]. Since our experiments showed that the uptake of glucose was very low, it is possible that a fraction of paramylon (β-1,3-glucan) is degraded into glucose-1-phosphate monomers that, upon isomerization, feed glycolysis.

Our data and those of others [13] allow us to conclude that in *D. papillatum*, the TCA cycle is not fed by pyruvate originating from glucose but by amino acids, which represent a major nutritional component not only of phagocytic heterotrophs, such as diplonemids, but also of the kinetoplastid parasites, including *Trypanosoma cruzi* and *T. brucei* [48, 49]. Amino acids constitute a scaffold for glucose monomers that may dimerize into trehalose, a highly abundant glucose derivate in *D. papillatum*, or alternatively polymerize into paramylon. Indeed, proline rather than glucose is a preferred precursor for trehalose synthesis [50]. The amino acid metabolism requires a robust transamination network that allows the transfer of the amino group to different acceptors. Indeed, we identified several transaminases in the transcriptome and proteome, mostly with the predicted organellar localization. We foresee that a targeted search would reveal even more of them.

Parasitic kinetoplastids exhibit numerous adaptations to different energy resources. During its life cycle in the tsetse fly vector, *T. brucei* relies on efficient catabolism of proline to succinate, acetate, and alanine as the main secreted end products, whereas in the blood of its vertebrate host, the plentiful glucose becomes the only usable carbon source [51]. Previous data signified the dominance of glucose synthesis via gluconeogenesis over its oxidation in glycolysis in *D. papillatum* [13]. We extended the experiments to hypoxia, having in mind the Pasteur effect, a general metabolic adaptation to anaerobiosis, in frame of which glucose uptake and oxidation are stimulated by low oxygen [52]. However, it seems not to operate in the studied protist, as oxygen has only a negligible effect on its glucose uptake. In cells cultivated in the oxygenized poor medium supplemented with ^14^C-glucose, lipids, saccharides, and FA became isotopically labeled, although when ^14^C-proline was provided under the same conditions, significantly more metabolites incorporated the label. Glucose is oxidized despite the absence of HK/GLK and PGI in the proteomic data (this study) and the lack of immunodetection of HK [13]. In *T. brucei*, HK and PFK are subject to allosteric regulation that possibly relates to the compartmentalization of glycolysis into glycosomes [53]. The constant HK activity in all tested *D. papillatum* samples points to the same way of regulation. Since the endogenous glucose may enter glycolysis as glucose-6-phosphate, the HK activity and glucose uptake are irrelevant for the evaluation of glycolytic rate. As in seawater glucose is present in varying concentrations, *D. papillatum* may occasionally meet conditions favorable for its uptake [54]. Although many marine protists do not encode glucose transporters, the *D. papillatum* transcriptome contains a sodium/glucose cotransporter. However, its involvement in the uptake of extracellular glucose should be confirmed experimentally, as it may as well participate in intracellular glucose distribution.

The identified opines and lactate may under the low-oxygen conditions play a role in NAD^+^ regeneration, consuming pyruvate for glycolysis demands [55]. Since opines do not change the intracellular pH and neutralize the cellular osmotic stress [19], in the ocean, their synthesis may be more advantageous than the production of lactate. Opines, which have the osmoprotective capacity [56], are not excreted and once environmental conditions change back to normoxia, they are conveniently reoxidized to pyruvate. Interestingly, previous studies showed OPDH to be substrate-specific, catalyzing dehydrogenation of a single amino acid [57, 58]. The presence of only one OPDH but several opines points to its ability to synthesize different types of opines. Indeed, the low affinity of OPDH for nonspecific substrates resulted in the synthesis of a small amount of various opines [59]. Curiously, our phylogenetic analysis suggests that *D. papillatum* and *E. gracilis* acquired their OPDHs by independent HGTs from different bacterial sources.

The MGTA and DGTA identified in *D. papillatum* belong to non-phosphorous, polar glycerolipids regarded to be analogous to phosphatidylcholines occurring in some microalgae, in contrast to the more abundant GTS analogues reported in bacteria, algae, fungi, and some land plants [60]. Although their biochemical and physiological role remains largely unknown, by analogy with the studied isomeric diacyl GTS analogues (DGTS) in microalgae such as *Nannochloropsis oceanica*, they are considered to be membrane lipids co-existing and/or replacing phosphatidylcholines in various stress situations, such as phosphate starvations or cold [61].

One of the characteristic features of euglenozoans is the polycistronic transcription [62, 63], and diplonemids are no exception [2]. Consequently, it is the proteome that informs us best about the metabolic pathways and their regulation under different environmental conditions. *D. papillatum* seems to survive hypoxia due to slower division and energy-saving inhibition of its metabolism. Moreover, this protist is equipped with enzymes for hypoxic metabolism needed in oxygen deficiency. Heterotrophic protists generally tolerate hypoxia very well [64], and this also applies to *D. papillatum*. However, in the nutrient-rich hypoxic conditions, the cell density remains stable, as the slow proliferation rate is balanced by the death rate.

Unusual metabolites identified by MS may shed further light on the role of diplonemids in the marine ecosystem. To our surprise, we have identified gonyol, which affects the marine sulfur cycle by modulating the release of methanethiol and dimethyl sulfide into the atmosphere [65]. The synthesis of gonyol was so far known only from photosynthesizing diatoms, dinoflagellates, and haptophytes [65] and, consequently, higher levels of the climate-influencing dimethyl sulfide occur in areas where an increased activity of phytoplankton was recorded [66]. Hence, diplonemids are the first heterotrophs known to produce not only gonyol but also trigonelline, so far considered to be plant-specific [67]. Since both metabolites serve as a carbon source for marine bacteria, diplonemids represent an important producer of bioavailable carbon and contribute to plankton fingerprints in the marine environment.

## Conclusions

The complexities of transcriptome, proteome, and metabolome reveal unusual flexibility of *D. papillatum* and likely other diplonemids, allowing them to survive under a wide range of conditions. Although not a true anaerobe, the extensive set of anaerobic enzymes allows the dissected flagellate to face nutrient and/or oxygen deprivation and even survive hypoxia, awaiting the improvement of conditions. *D. papillatum* seems to gain flexibility by retaining alternative metabolic pathways and to be able to switch among them efficiently.

## Methods

### Cell cultivation

*D. papillatum* ATCC 50162 was grown axenically in vented flasks at 15 °C in a seawater-based nutrient-rich (R) medium (1 g/l tryptone and 1% (v/v) FBS). To obtain nutrient-poor (P) conditions (0.01 g/l tryptone, 0.001% lysogeny broth, and 1% (v/v) horse serum), cells were inoculated from a stock culture from the R medium. In parallel, cells were placed into Oxoid AnaeroJar (Thermo Fisher Scientific) for 72 h to generate hypoxia in both media.

To assess growth, cells cultured in either the P or R medium were harvested, stained with CellTrace CFSE Cell Proliferation Kit (Invitrogen) according to the manufacturer protocol, resuspended into the respective medium at a concentration 5 × 10^4^ ml^−1^, and subjected to either aerobic or hypoxic conditions for 72 h. Fluorescence intensity and cell concentrations were measured by flow cytometry using FACS Canto II (BD Biosciences) operating on medium throughput for 20 s. To examine cell morphology, live cells from 72-h-old cultures were observed under the Olympus BX53 microscope equipped with differential interference contrast. Images were taken with a DP72 digital camera at 1600 × 1200-pixel resolution using CellSens software v1.11 (Olympus) and processed with ImageJ v1.51 software.

### Transcriptome analysis

To confirm prior to RNA isolation that the culture is monoeukaryotic, the V4 motif of the 18S rRNA gene was amplified and sequenced, and its identity confirmed the sole presence of *D. papillatum*. Total RNA was isolated from cells grown in R and P media in normoxia and hypoxia using a protocol described previously [68]. RNA-Seq polyA libraries were sequenced using Illumina paired-end 150-bp technology. Adapter sequences and the poor-quality regions were trimmed using Trimmomatic v0.39 [69] with the default settings, and the cleaned reads were assembled in rnaSPAdes v3.14 [70]. To obtain a representative set of transcripts, all 12 read sets were assembled together. To reduce the risk of bacterial contamination, all predicted transcripts were screened against NCBI non-redundant database using DIAMOND [71]. Only transcripts with at least one eukaryotic HSP (High-scoring Segment Pair) among the top three scoring hits were considered for further analyses. Protein sequences were predicted using TransDecoder v5.2 (https://github.com/TransDecoder/TransDecoder) under the default settings and were annotated in OmicsBox v1.4 [72] and KofamKOALA [73] Web service. Transcript abundance was estimated using Salmon [74]. For each pair of experimental conditions, differentially expressed transcripts were estimated from the abundance matrices using both edgeR v3.32.1 [75] and DESeq2 v1.30.1 [76] R modules. Only transcripts assigned as differentially expressed (false discovery rate [FDR] < 1e−3 and log_2_fold > 2) by both methods were considered for further analyses.

### Sequence searches and phylogenetic analysis

Sequences of interest were searched in the *D. papillatum* transcriptome by BLAST v2.2.31+ [77] using *T. brucei* and *E. gracilis* sequences as queries. Protein domains were predicted by InterProScan [78] implemented in the Geneious Prime v2020.2.3 software [79].

The OPDHs of *D. papillatum* and *E. gracilis* were added to a previously published dataset [19] and homologues identified in BLAST searches against the NCBI non-redundant database. The dataset was aligned using MAFFT v7.458 under L-INS-i strategy [80], with poorly aligned positions removed by trimAl v1.4 (-gt 0.8) [81]. The final alignment contained 247 taxa and 251 positions. The maximum likelihood phylogenetic tree was inferred in IQ-TREE v1.6.12 [82] under the LG+G4 model (determined as the best-fitting model according to Bayesian information criterion) and 1000 ultrafast bootstrap replicates.

### Localization predictions

Localization of selected proteins was determined using TargetP v2 [83], NommPred [84], MultiLoc2 [85], and MitoFates [86] prediction tools. NommPred was used in the mitochondria and Trypanosomatida settings, as diplonemids harbor a mitochondrion and are sister to kinetoplastids. Since *D. papillatum* does not possess a plastid, the plant setting from MultiLoc2 and MitoFates was omitted, and only fungal and animal/metazoan settings were used. To identify potential glycosomal proteins, sequences were searched for the presence of peroxisomal targeting signals (PTS) using an in-house python script (https://github.com/kikinocka/ngs/blob/master/py_scripts/pts_search.py). Based on kinetoplastid glycosomal proteins [87], sequences [SAGCNP]-[RHKNQ]-[LIVFAMY]$ and ˆM-x(0,20)-[RK]-[LVI]-x5-[HKQR]-[LAIVFY] were used to search for C-terminal PTS1 and N-terminal PTS2, respectively.

### Identification and label-free quantification of proteins by mass spectrometry

For protein quantification by liquid chromatography-coupled mass spectrometry, 100 μg of proteins obtained from biological triplicates cultivated in R and P media in aerobic (+) and hypoxic (-) conditions for 72 h were used. Cell pellets were dissolved in 8 M urea, reduced by 5 mM dithiothreitol, and alkylated with 40 mM iodoacetamide. The reaction was quenched by additional 5 mM dithiothreitol. After dilution in 3 volumes of 50 mM triethylammonium bicarbonate buffer (pH 8), the proteins were digested overnight by sequencing grade trypsin (1:60, w/w) (Promega). Peptide solution, acidified by 0.5% trifluoroacetic acid, was clarified by centrifugation and purified on custom-made microtips filled with LiChroprep RP-18 25–40-μm particles (Merck-Millipore). Upon vacuum evaporation in Concentrator plus (Eppendorf), the peptides were dissolved in 0.1% trifluoroacetic acid and 2% acetonitrile, and their concentration was determined by Pierce Quantitative Fluorometric Peptide Assay (Thermo Fisher Scientific).

Next, 500 ng of purified peptides per sample was loaded onto a trap column (PepMap100 C18, 300 μm × 5 mm, 5-μm particle size) (Dionex) and separated with an EASY-Spray C18 analytical column having integrated nanospray emitter (75 μm × 500 mm, 5-μm particle size) (Thermo Fisher Scientific) on Ultimate 3000 RSLCnano system (Dionex) in a 120-min gradient (3–43% B), concave curve type 7, and flow rate 250 nl/min. Two mobile phases were used—0.1% formic acid (v/v) and 80% ACN (v/v) with 0.1% formic acid. Eluted peptides were sprayed directly into Orbitrap Elite mass spectrometer (Thermo Fisher Scientific), equipped with EASY-Spray ion source, and spectral datasets were collected in the data-dependent mode using Top15 strategy for the selection of precursor ions [88]. Precursors were measured in the mass range 300–1700 *m*/*z* with resolution 120,000, and fragments were obtained by the HCD mechanism with normalized collision energy 25 and resolution 15,000. Each of the three biological replicates was analyzed in three technical replicates.

Obtained datasets were processed by MaxQuant v1.6.17.0 [89] with a built-in Andromeda search engine and the following parameters: (i) carbamidomethylation (C) as permanent and oxidation (M) as variable modifications; (ii) 20 ppm peptide tolerance in the first search, 4.5 ppm in the main search upon recalibration, and 20 ppm fragment tolerance; (iii) 1% peptide and protein false discovery rates based on reverse decoy database search; (iv) engaged “match between the runs” feature and label-free quantification. The label-free quantification (LFQ intensities) relied on sums of precursor ion intensities of unique proteotypic peptides upon normalization by the median distribution of all ions. The search was performed against de novo assembled transcriptome-derived protein sequences (87,769 sequences). Technical replicates were combined in a single sample for increasing depth and completeness of data.

The statistical analysis was performed using Perseus v1.6.15.0. Output proteinGroup table from MaxQuant was filtered for the reverse proteins, the contaminants, and the low confidence proteins identified only by the site. After log_2_ transformation of the LFQ intensities, only proteins with two and more valid values in at least one experimental group were retained. Consequently, the missing values were imputed from the normal distribution. Principal component analysis was used to evaluate sources of variability among samples and replicates. Next, ANOVA was performed with Benjamini-Hochberg correction for multiple testing with a *q*-value threshold at 0.01. For pairwise comparisons, post hoc Tukey’s test was used at *P* ≤ 0.01. Additionally, differentially abundant proteins were filtered on effect size, at least 1-fold of log_2_-transformed ratio. Hierarchical clustering was performed on *Z*-score-normalized averages of LFQ intensities for revealing protein abundance trends within specific functional groups of proteins across experimental conditions.

### LC-HRMS analysis of metabolome

#### Solvents and reagents

The deionized water was prepared using a Direct Q 3UV purification system (Merck). Methanol and acetonitrile (Optima grade) were obtained from Thermo Fisher Scientific, ammonium carbonate; 25% ammonia solution, 4-fluorophenylalanine, 1C6-glucose-6-phosphate, and 2-dipalmitoyl-*sn*-glycero-3-O-4′-[N,N,N-trimethyl(d9)]-homoserine (d9-DGTS) from Merck; and hexakis(2,2-difluoroethoxy)phosphazene and tris(trifluoromethyl)-1,3,5-triazene from Apollo Scientific.

#### Sample preparation

For the LC-HRMS analysis performed in triplicates, cells were cultured as described above. For each replicate, 6 × 10^6^ cells were pelleted, rinsed with a salt solution (450 mM NaCl; 10 mM KCl; 9 mM CaCl_2_; 30 mM MgC_12_·6H_2_O; 16 mM MgSO_4_·7H_2_O) mimicking seawater. The enzymatic activity was immediately stopped with an ice-cold extraction medium with internal standard (100 μl MeOH: ACN: H_2_O [2:2:1 v/v/v] plus 4-fluorophenylalanine [1 nmol/ per sample]). Cell suspension was homogenized by an ultrasonic bath (5 min, 0 °C) properly mixed and sonicated under the same conditions. The mixture was then centrifuged at 4490 *g* for 10 min at 4 °C and the supernatant was separated. The process of homogenization (extraction) and centrifugation was repeated (100 μl MeOH: ACN: H_2_O [2:2:1 v/v/v]). Following second centrifugation, the supernatant was collected and filtered by a 0.2-μm PVDF mini-spin filter (HPST) at 6080 *g* for 10 min at 5 °C. Finally, the mini-spin filter was rinsed with 20 μl of the extraction medium. The filtered supernatant was evaporated in a vacuum concentrator (Jouan RC 10.10 and RCT 60). Each sample residue was reconstituted in 50 μl 50% acetonitrile, thoroughly mixed (30 s), and placed in the ultrasonic bath (5 min). The prepared sample was directly measured by LC-HRMS.

#### LC-HRMS analysis and data processing

A high-resolution Orbitrap Q Exactive Plus mass spectrometer coupled to a Dionex Ultimate 3000 liquid chromatograph and a Dionex open autosampler (Thermo Fisher Scientific) was used for metabolite profiling and quantitative analysis. Metabolites were separated on SeQuant ZIC-pHILIC 5-μm polymer 150 mm × 4.6 mm PEEK-coated HPLC column (Merck) with a mobile phase flow rate 450 μl/min, and the injection volume 5 μl, column temperature 35 °C. The mobile phase was as follows: A = acetonitrile, B = 20 mmol/l aqueous ammonium carbonate (pH 9.2, adjusted by NH_4_OH), gradient: 0 min, 20% B; 20 min, 80% B; 20.1 min, 95% B; 23.3 min, 95% B; 23.4 min, 20% B; 30.0 min 20% B.

Full-scan HRMS positive ion and negative ion mass spectra were recorded in a separate run in a mass range 70–1050 Da at 70,000 resolution (200 *m*/*z*). The Q-Exactive settings were as follows: scan rate ± 3 Hz, 3 × 106 automatic gain control (AGC) target, maximum ion injection time (IT) 100 ms, ion source parameters ± 3000 kV spray voltage, 350 °C capillary temperature, sheath gas at 60 au, aux gas at 20 au, spare gas at 1 au, probe temperature 350 °C, and S-Lens level at 60 au. For accurate mass measurements, lock masses 622.0290 *m*/*z* and 301.9981 *m*/*z* were used for the positive and negative ion detection mode, respectively. The data were processed using an Xcalibur v4.0 software (Thermo Fisher Scientific) and an in-house built KEEG Metabolite Mapper platform generating HRMS extracted peak features and equipped with an internal metabolite database. The distinct HRMS signals of small ionic metabolites were detected, matched with the in-house metabolomic library containing > 2500 metabolites and available chemical standards, and unambiguously identified in most cases. The raw data are presented in Additional file 4: Table S2 without any further statistical modifications.

### Enzymatic assays

For SDH enzymatic assays, mitochondria isolated by hypotonic lysis from 5 × 10^8^ cells were lysed on ice for 1 h in 2% (w/v) dodecyl-maltoside and 0.4 M aminocaproic acid. Upon centrifugation at 24,400 *g* for 30 min at 4 °C, the supernatant was used for activity determination as described previously [90]. Aliquot was pre-incubated with SDH buffer (25 mM KPi, pH 7.2; 5 mM MgCl_2_; 20 mM sodium succinate) at 25 °C for 10 min. Next, antimycin A, rotenone, KCN, and 2,6-dichlorophenolindophenol (DPIP) were separately added to a final concentration of 1.8 mM, 5 mM, 2 mM, and 50 μM, respectively. The reaction itself was started by the addition of coenzyme Q_2_ to a final concentration of 65 μM and monitored at 600 nm for 5 min.

To assess hexokinase activity, 5 × 10^8^ cells were lysed on ice for 1 h in 2% (w/v) dodecyl-maltoside and 0.4 M aminocaproic acid. Upon centrifugation at 24,400 *g* for 30 min at 4 °C, the supernatant was used for activity determination. Hexokinase activity was measured in 1-ml reaction buffer containing 50 mM Tris-HCl pH 8.0, 13 mM MgCl_2_, 0.55 mM ATP, 0.22 mM NAD^+^, and 0.1 M glucose. The reaction was started by the addition of 3 U of glucose-6-phosphate dehydrogenase and monitored at 340 nm for 3 min. Protein concentration was assessed by Bradford assay [91]. All measurements were carried out in biological triplicates.

### Immunodetection

The total cell (50 μg) or mitochondrial lysates (30 μg) were resolved on a 10% SDS-PAGE gel, followed by a transfer onto nitrocellulose membrane under wet blot conditions at 20 mA overnight. Antibodies against α-tubulin and glycosomal enzymes PGI and ENO were used as described elsewhere [13]. For detection of flavine subunit of the SDH complex, polyclonal anti-sdh66 antibodies were used at 1:1000 dilution.

### Protein profile

To assess the difference in protein profiles upon cultivation in R+, R-, P+, and P- conditions, cells well obtained from 72-h cultures and washed in sea salt solution, and upon cell lysis, proteins were separated on a 10% SDS-PAGE gel stained with 0.25% (w/v) Coomassie Brilliant Blue R-250 in 10% (v/v) acetic acid and 30% (v/v) methanol.

### Labeling with ^14^C[U]-proline or ^14^C[U]-glucose

Cultures at a concentration of 5 × 10^5^ cells/ml were inoculated into 3 ml of P medium supplemented with ^14^C[U]-proline or ^14^C[U]-glucose at a final activity concentration of 1 μCi/ml and cultivated for 24 h in a stationary phase. Next, cells were harvested at 1000 *g* for 10 min, washed twice in sea salt solution, and subjected to acetone sonication. The pellets and acetone supernatants were processed for saccharides, lipids, fatty acids, and protein isolations as described elsewhere [92, 93]. Radioactivity was visualized by the exposure of the TLC plates to Kodak X-Omat AR film at −70 °C.

Five × 10^5^ cells were inoculated into 1 ml of R and P media on a 24-well plate. Each medium was supplemented with ^14^C[U]-glucose at a final activity concentration 1 μCi/ml, and the cells were cultivated stationary for 24 h in the presence or absence of oxygen. Homogenates were prepared as described above and subjected to liquid scintillation spectrometry (Perkin Elmer), quantified as disintegrations per min (DPM), and evaluated in absolute counts where the amount of DPM in cell culture was taken as maximum. To assess the glucose uptake, we proceeded analogously after 12-h incubation of cells in sea salt solution supplemented with the ^14^C[U]-glucose at a final activity concentration 1 μCi/ml.

## Supplementary Information

**Additional file 1: Fig. S1.** LC-HRMS metabolic profiles. Characteristic (**a**) posESI HRMS and (**b**) negESI HRMS metabolic signatures for the *D. papillatum* cell extracts grown in rich normoxic (R+), rich hypoxic (R-), nutrient poor normoxic (P+), and nutrient poor hypoxic (P-) conditions. 3-Ala, 3-alanine; Ac-Carn, acyl-carnitine; Acyl-Me-Tau, acyl-methyl-taurines (taurates); Ade, adenine; Ado, adenosine; Ala, alanine; Arg, arginine; Arg, arginine; DMSP, 3-dimethylsulfoniopropionate; FA, fatty acid; GMAB, glyceromethyl-3-alanine betaine; Gln, glutamine; GPC, glycerophosphocholine; GSH, glutathione; HRMS, high resolution mass spectrometry; Ile, isoleucine; Betaine LP, betaine-lipids; Lys, lysine; Mal, malate; MeTau, N-methyltaurine; negESI, negative electrospray ionization; Orn, ornithine; PA, phosphatidic acid; PC, phosphatidylcholine; PE, phosphatidylethanolamine; Pro, proline; posESI, positive electrospray ionization; PS, phosphatidylserine; Tau, taurine; Tre, trehalose; Val, valine. **Fig. S2.** Sequences of phosphofructokinase (PFK) and pyrophosphate-fructose phosphotransferase (PFP)**.** (**a**) Alignment of PFK1 shows that the sequence previously identified [13] was truncated at its N-terminus. Moreover, predicted protein domains classify it as PFP rather than PFK. (**b**) Alignment of PFK2 identified in this and previous study [13]. (**c**) Sequence of PFP identified only in this study. Color boxes correspond to predicted protein domains as in different databases as explained in graphical legend. **Fig. S3.** ^14^C-proline (Pro) and ^14^C-glucose (Glu) uptake in *D. papillatum***.** (**a**) Cultures of 5 × 10^6^ cells were fed with ^14^C-Pro and ^14^C-Glu in poor medium under aerobic conditions. Shown are autoradiograms of separated proteins, monosaccharides, lipids, and fatty acids. (**b**) Cells were cultivated for 24 h in the presence of radioactive glucose in rich (R) and poor (P) medium under aerobic (+) and hypoxic (-) conditions. (**c**) Cells were placed into sea salt solution supplemented with radioactive glucose and cultured for 12 h. The amount of isotope was determined with scintillation counter as disintegrations per minute (DPM) in 1 ml of growth medium containing 5 × 10^6^ cells and cellular lysates. **Fig. S4.** Heatmaps of proteins involved in transcription (a), translation (b), and detoxifying systems (c). Abbreviations: rich medium (R), poor medium (P), aerobic conditions (+) hypoxic conditions (-). **Fig. S5.** Phylogenetic analysis of *D. papillatum* opine dehydrogenase. The Maximum Likelihood phylogenetic tree was estimated under the LG + G4 model (chosen as best-fitting model) with ultrafast bootstrapping. Support values are shown when ≥75%.

**Additional file 2: File S1.** Original gel of Coomassie-stained SDS-PAGE**.** Lanes 2-5 correspond to protein profiles shown in Fig. 1D. **File S2.** Original autoradiograms. Lanes 3-4 of ^14^C-labeled proteins (**a**), monosaccharides (**b**), lipids (**c**), and fatty acids (**d**) are shown in Additional file 1: Fig. S3A. **File S3.** Original western blots**.** Lanes 6-9, 1-4, 2-5, and 2-5 of immunodetected phosphoglucose isomerase (**a**), enolase (**b**), α-tubulin (**c**), and succinate dehydrogenase subunit I (**d**), respectively, are shown in Fig. 5.

**Additional file 3: Table S1.** List of enzymes identified in the transcriptome and proteome of *D. papillatum*. “Predicted localization” in column L summarizes all predicted localizations obtained using an in-house python script and four different tools, three of them in two different settings. Abbreviations used for cellular localization (columns D-L), transcriptomic (columns M-O) and proteomic (columns P-U) data are explained below the table. Values of transcriptomic and proteomic ratios are log_2_ transformed fold change. ANOVA was performed with Benjamini-Hochberg correction for multiple testing with a pValue threshold at 0.01. For pairwise comparisons, post hoc Tukey’s test was used at p ≤ 0.01. Differentially abundant proteins were filtered on effect size, at least 1-fold of log_2_ transformed ratio. Abbreviations in column headings: rich medium (R), poor medium (P), aerobic conditions (+) hypoxic conditions (-).

**Additional file 4: Table S2.** List of metabolites identified in *D. papillatum*. A comprehensive metabolite list covering all important compounds detected in the *D. papillatum* metabolome, their analytical characteristics including identification tools, metabolic trend changes in four compared conditions (R+, R-, P+, P-). The Metabolite Mapper generated metabolomic data mining presented as a series of sheets comprising a metabolite list (S3a), and boxplot diagrams of each detected metabolite arranged accordingly to a particular metabolic pathway (S3b-S3j). The up- and downregulated significant changes between groups are highlighted by blue and pink backgrounds, respectively. Thresholds: Log_2_-fold change ≤2, and p-value ≤0.05. For p-value calculation, the two-sample unequal variance test with symmetrical distribution was used.

**Additional file 5: Data S1.** KEGG maps.

**Additional file 6: Data S1.** Identification and annotation of the uncommon metabolites of *D. papillatum* by LC-HRMS analysis.

## Data Availability

All data supporting the conclusions of this article are included within the article and its additional files. The raw RNA-Seq reads and representative transcriptome assembly are available at NCBI under the BioProject PRJNA741790 [94]. The mass spectrometry proteomics data have been deposited to the ProteomeXchange Consortium via the PRIDE [95] partner repository under the dataset identifier PXD025411 (doi.10.6019/PXD025411) [96].

## Abbreviations

ACC: Acetyl-CoA carboxylase
ACL: ATP-citrate lyase
ACO: Aconitase
ACS: Acyl-CoA synthetase
CoA: Coenzyme A
DGTA: Diacyl glycerol-3-trimethyl alanine
DGTS: Diacyl glycerol-N-trimethyl homoserine
DHAP: Dihydroxyacetone phosphate
DPM: Disintegrations per minute
ECH: Enoyl-CoA hydratase
ENO: Enolase
FA: Fatty acid
FAS-I: Fatty acid synthase type I
FAS-II: Fatty acid synthase type II
FBPase: Fructose-1,6-bisphosphatase
FBS: Fetal bovine serum
FDR: False discovery rate
FRD: Fumarate reductase
G3P: Glycerol 3-phosphate
G3PDH: Glycerol 3-phosphate dehydrogenase
GAPDH: Glyceraldehyde-3-phosphate dehydrogenase
GLK: Glucokinase
GPase: Glucose-6-phosphatase
GPDH: Glucose-6-phosphate dehydrogenase
GTS: Glycerol-N-trimethyl homoserine
HADC: 3-Hydroxyacyl-CoA dehydrogenase
HK: Hexokinase
I-IV: Respiratory complex I-IV
IDH: Isocitrate dehydrogenase
KACT: 3-Ketoacyl-CoA thiolase
LC-HRMS: Liquid chromatography-high-resolution mass spectrometry
LDH: Lactate dehydrogenase
LFQ: Label-free quantification
MDH: Malate dehydrogenase
ME: Malic enzyme
MGTA: Monoacyl glycerol-3-trimethyl alanine
MQ: Menaquinone
MS: Mass spectrometry
NDH2: NADH dehydrogenase
OGDC: 2-Oxoglutarate decarboxylase
OPDH: Opine dehydrogenase
OXPHOS: Oxidative phosphorylation
P+: Poor aerobic conditions
P-: Poor hypoxic conditions
PC: Pyruvate carboxylase
PDH: Pyruvate dehydrogenase complex
PEPCK: Phosphoenolpyruvate carboxykinase
PFK: Phosphofructokinase
PFP: Pyrophosphate fructose-6-phosphate 1-phosphotransferase
PGI: Phosphoglucose-6-isomerase
PGK: Phosphoglycerate kinase
PK: Pyruvate kinase
PNO: Pyruvate:NADP^+^ oxidoreductase
PPP: Pentose phosphate pathway
ProDH: Proline dehydrogenase
R+: Rich aerobic conditions
R-: Rich hypoxic conditions
SDH: Succinate dehydrogenase/respiratory complex II
SSDH: Succinate-semialdehyde dehydrogenase
TCA: Tricarboxylic acid cycle
UQ: Ubiquinone
