# Supplementary material for: Highly flexible metabolism of the marine euglenozoan protist Diplonema papillatum
Source: BMC Biol. 2021 Nov 24;19:251. doi: 10.1186/s12915-021-01186-y (PMC8611851; doi:10.1186/s12915-021-01186-y)
Supplement: Supplementary file 1 — Additional file 1: Fig. S1. LC-HRMS metabolic profiles. Characteristic (a) posESI HRMS and (b) negESI HRMS metabolic signatures for the D. papillatum cell extracts grown in rich normoxic (R+), rich hypoxic (R-), nutrient poor normoxic (P+), and nutrient poor hypoxic (P-) conditions. 3-Ala, 3-alanine; Ac-Carn, acyl-carnitine; Acyl-Me-Tau, acyl-methyl-taurines (taurates); Ade, adenine; Ado, adenosine; Ala, alanine; Arg, arginine; Arg, arginine; DMSP, 3-dimethylsulfoniopropionate; FA, fatty acid; GMAB, glyceromethyl-3-alanine betaine; Gln, glutamine; GPC, glycerophosphocholine; GSH, glutathione; HRMS, high resolution mass spectrometry; Ile, isoleucine; Betaine LP, betaine-lipids; Lys, lysine; Mal, malate; MeTau, N-methyltaurine; negESI, negative electrospray ionization; Orn, ornithine; PA, phosphatidic acid; PC, phosphatidylcholine; PE, phosphatidylethanolamine; Pro, proline; posESI, positive electrospray ionization; PS, phosphatidylserine; Tau, taurine; Tre, trehalose; Val, valine. Fig. S2. Sequences of phosphofructokinase (PFK) and pyrophosphate-fructose phosphotransferase (PFP). (a) Alignment of PFK1 shows that the sequence previously identified [13] was truncated at its N-terminus. Moreover, predicted protein domains classify it as PFP rather than PFK. (b) Alignment of PFK2 identified in this and previous study [13]. (c) Sequence of PFP identified only in this study. Color boxes correspond to predicted protein domains as in different databases as explained in graphical legend. Fig. S3. 14C-proline (Pro) and 14C-glucose (Glu) uptake in D. papillatum. (a) Cultures of 5 × 10^6^ cells were fed with 14C-Pro and 14C-Glu in poor medium under aerobic conditions. Shown are autoradiograms of separated proteins, monosaccharides, lipids, and fatty acids. (b) Cells were cultivated for 24 h in the presence of radioactive glucose in rich (R) and poor (P) medium under aerobic (+) and hypoxic (-) conditions. (c) Cells were placed into sea salt solution supplemented with radioactive g [file 12915_2021_1186_MOESM1_ESM.pdf]

Highly flexible metabolism of the marine euglenozoan protist *Diplonema papillatum*

Ingrid Škodová-Sveráková[1,2,#,*], Kristína Záhonová[1,3,#], Valéria Juricová[1,4], Maksym Danchenko[5], Martin Moos[6], Peter Baráth[5,7], Galina Prokopchuk[1], Anzhelika Butenko[1,8], Veronika Lukáčová[7], Lenka Kohútová[5], Barbora Bučková[2], Aleš Horák[1,4], Drahomíra Faktorová[1,4], Anton Horváth[2], Petr Šimek[6], and Julius Lukeš[1,4,*]

[1] Institute of Parasitology, Biology Centre, Czech Academy of Sciences, České Budějovice (Budweis), Czech Republic

[2] Faculty of Natural Sciences, Comenius University, Bratislava, Slovakia

[3] Faculty of Science, Charles University, BIOCEV, Vestec, Czech Republic

[4] Faculty of Sciences, University of South Bohemia, České Budějovice (Budweis), Czech Republic

[5] Institute of Chemistry, Slovak Academy of Sciences, Bratislava, Slovakia

[6] Institute of Entomology, Biology Centre, Czech Academy of Sciences, České Budějovice (Budweis), Czech Republic

[7] Medirex Group Academy, n.o., Trnava, Slovakia

[8] Faculty of Science, University of Ostrava, Ostrava, Czech Republic

[#] These authors contributed equally to this work

[*]Corresponding authors: jula@paru.cas.cz; skodovaister@gmail.com

Additional file 1: Fig. S1-S5

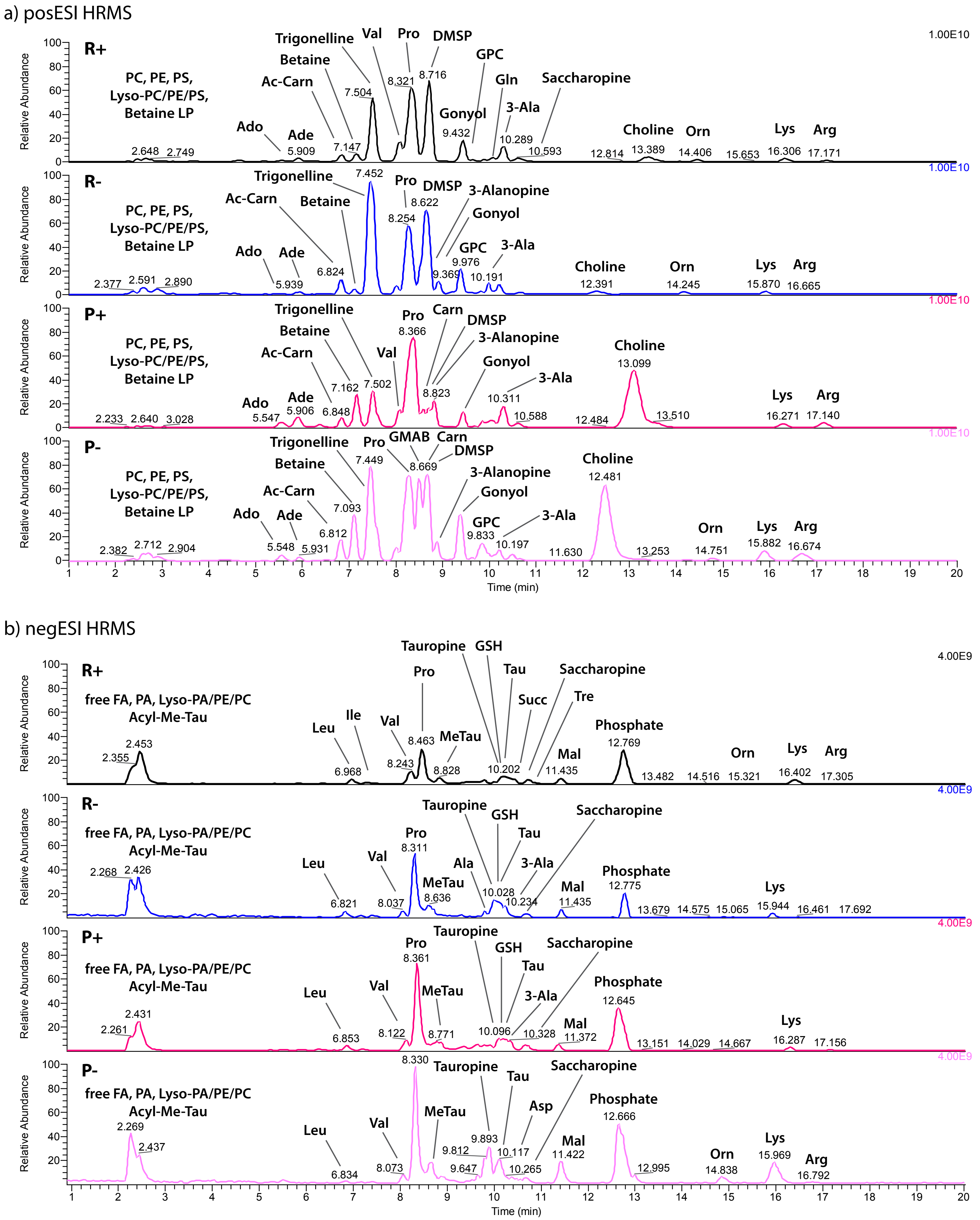

Fig. S1. LC-HRMS metabolic profiles. Characteristic (**a**) posESI HRMS and (**b**) negESI HRMS metabolic signatures for the *D. papillatum* cell extracts grown in rich normoxic (R+), rich hypoxic (R-), nutrient poor normoxic (P+), and nutrient poor hypoxic (P-) conditions. 3-Ala, 3-alanine; Ac-Carn, acylcarnitine; Acyl-Me-Tau, acyl-methyl-taurines (taurates); Ade, adenine; Ado, adenosine; Ala, alanine; Arg, arginine; Arg, arginine; DMSP, 3-dimethylsulfoniopropionate; FA, fatty acid; GMAB, glyceromethyl-3-alanine betaine; Gln, glutamine; GPC, glycerophosphocholine; GSH, glutathione; HRMS, high resolution mass spectrometry; Ile, isoleucine; Betaine LP, betaine-lipids; Lys, lysine; Mal, malate; MeTau, N-methyltaurine; negESI, negative electrospray ionization; Orn, ornithine; PA, phosphatidic acid; PC, phosphatidylcholine; PE, phosphatidylethanolamine; Pro, proline; posESI, positive electrospray ionization; PS, phosphatidylserine; Tau, taurine; Tre, trehalose; Val, valine.

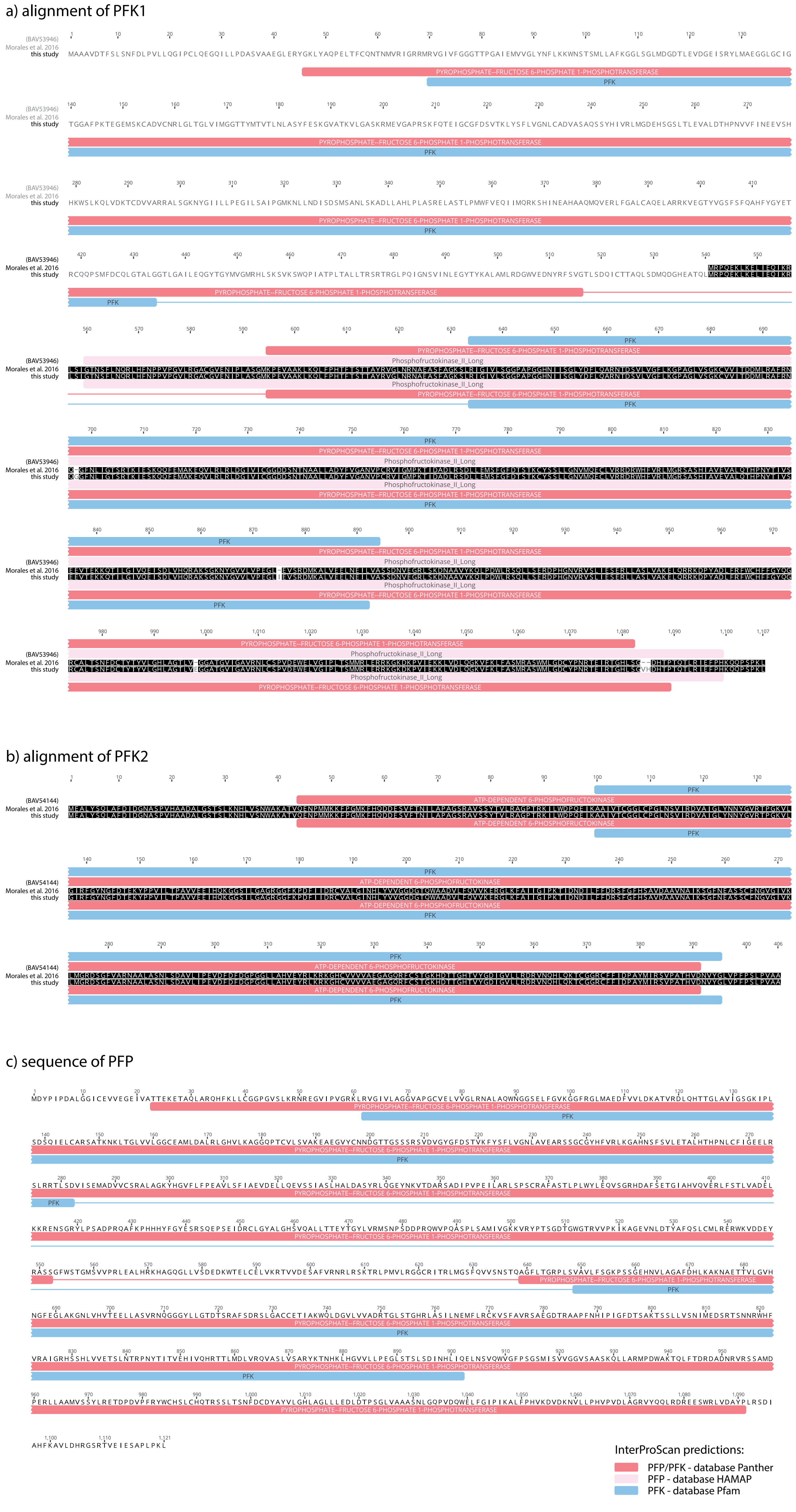

Fig. S2. Sequences of phosphofructokinase (PFK) and pyrophosphate-fructose phosphotransferase (PFP). (**a**) Alignment of PFK1 shows that the sequence previously identified [13] was truncated at its N-terminus. Moreover, predicted protein domains classify it as PFP rather than PFK. (**b**) Alignment of PFK2 identified in this and previous study [13]. (**c**) Sequence of PFP identified only in this study. Color boxes correspond to predicted protein domains as in different databases as explained in graphical legend.

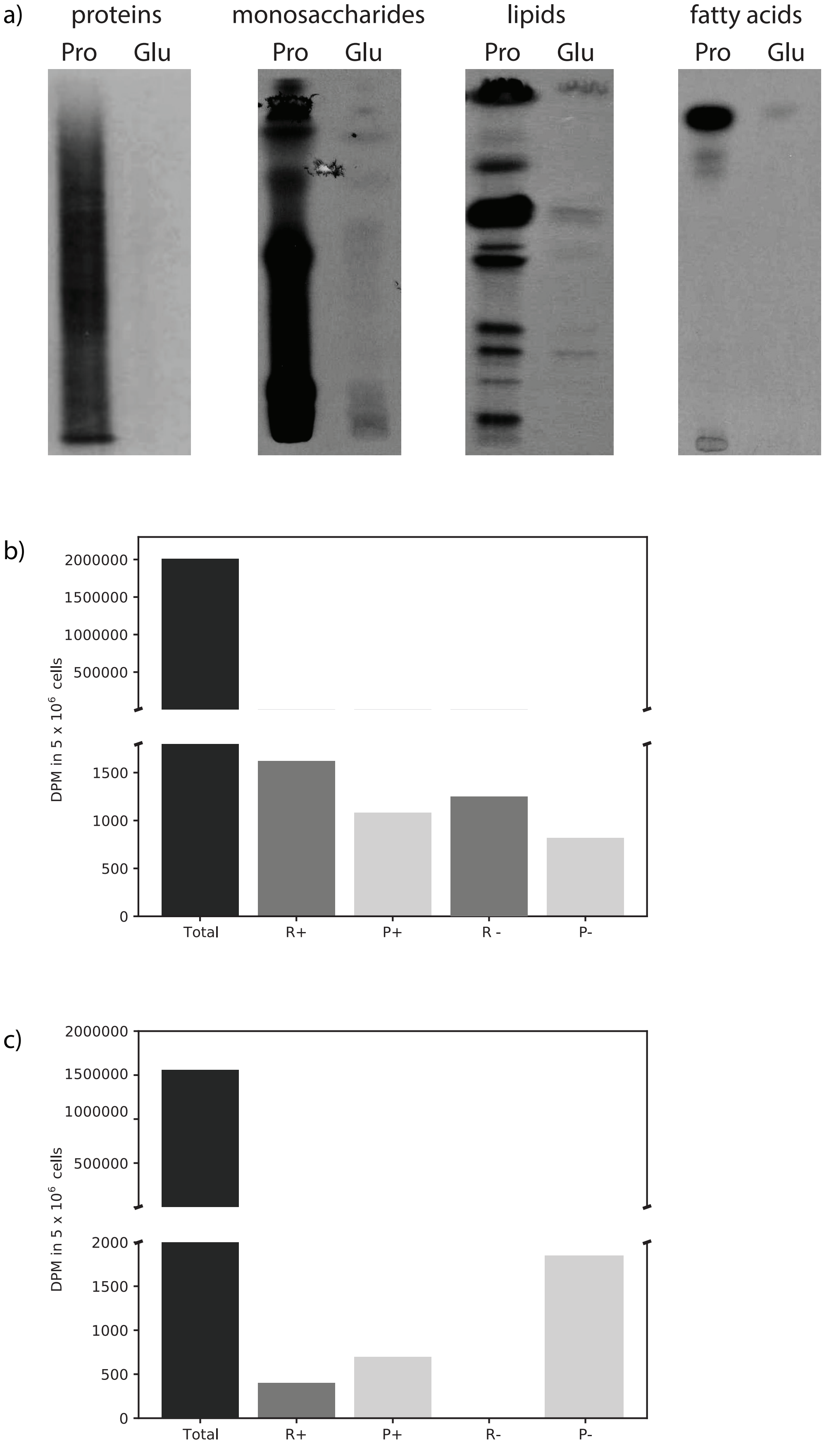

Fig. S3. ^{14}C-proline (Pro) and ^{14}C-glucose (Glu) uptake in D. papillatum. (**a**) Cultures of 5 x 10^6 cells were fed with ^{14}C-Pro and ^{14}C-Glu in the poor medium under aerobic conditions. Shown are autoradiograms of separated proteins, monosaccharides, lipids, and fatty acids. (**b**) Cells were cultivated for 24 h in the presence of radioactive glucose in rich (R) and poor (P) medium under aerobic (+) and hypoxic (-) conditions. (**c**) Cells were placed into sea salt solution supplemented with radioactive glucose and cultured for 12 h. The amount of isotope was determined with scintillation counter as disintegrations per minute (DPM) in 1 ml of growth medium containing 5 x 10^6 cells and cellular lysates.

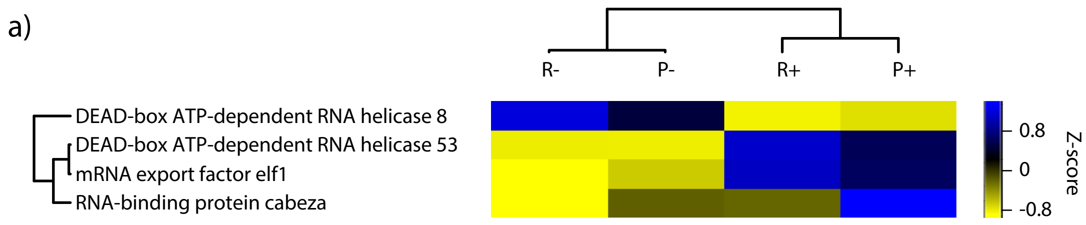

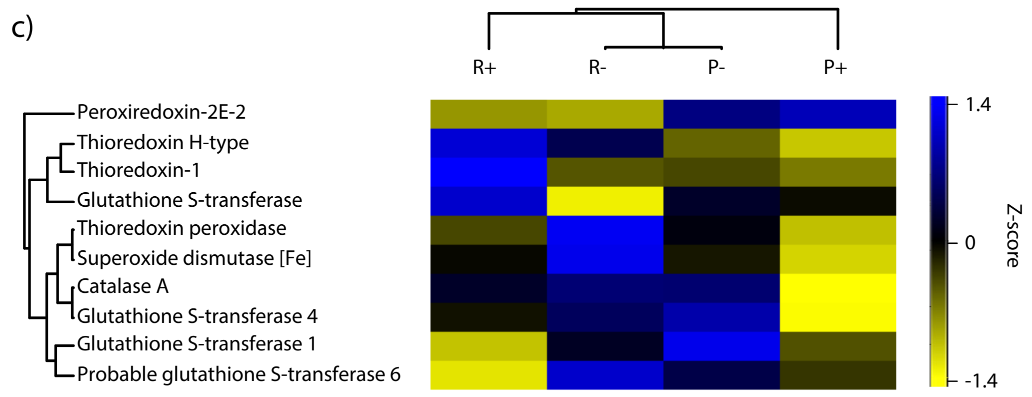

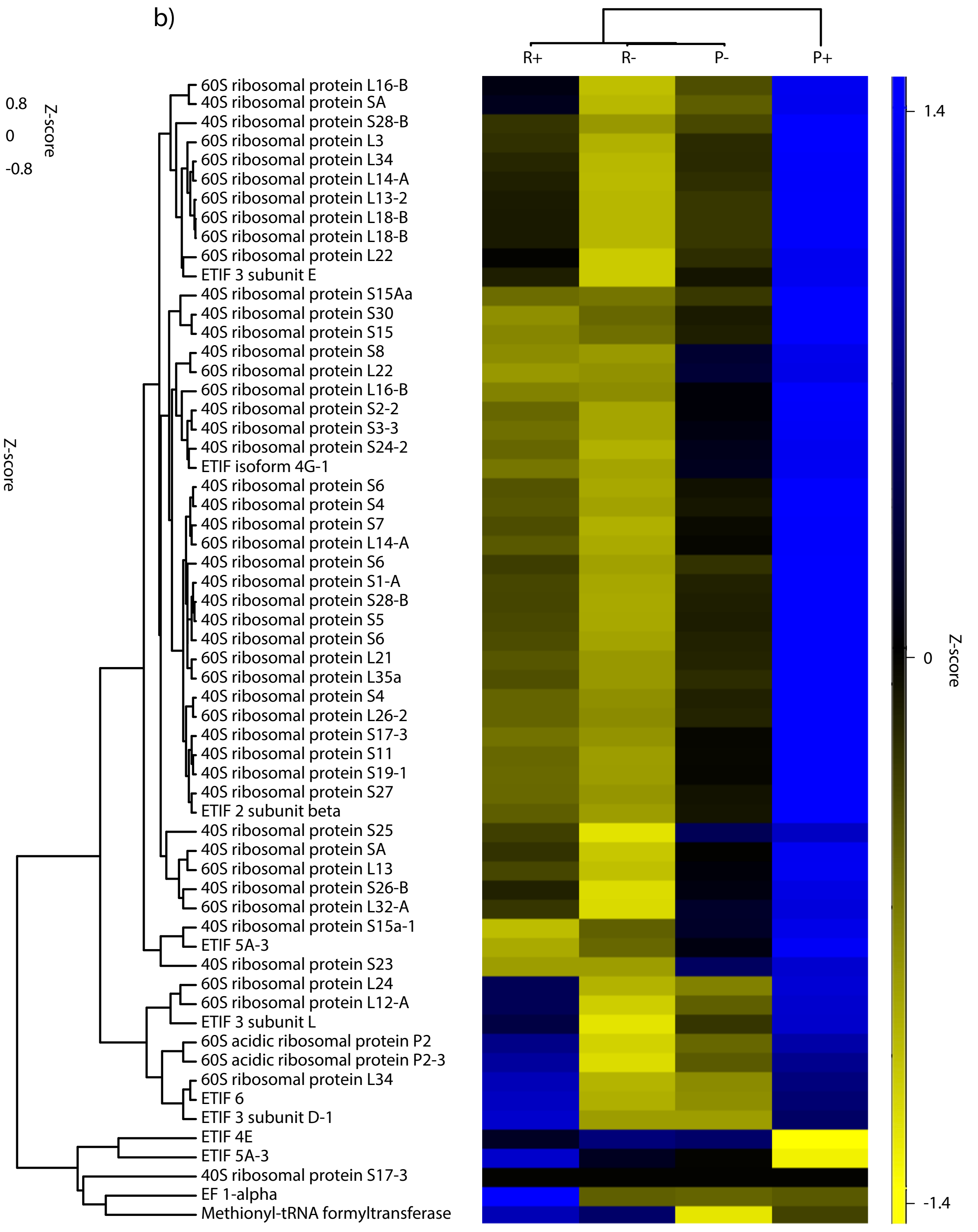

Fig. S4. Heatmaps of proteins involved in transcription (a), translation (b), and detoxifying systems (c). Abbreviations: rich medium (R), poor medium (P), aerobic conditions (+) hypoxic conditions (-).

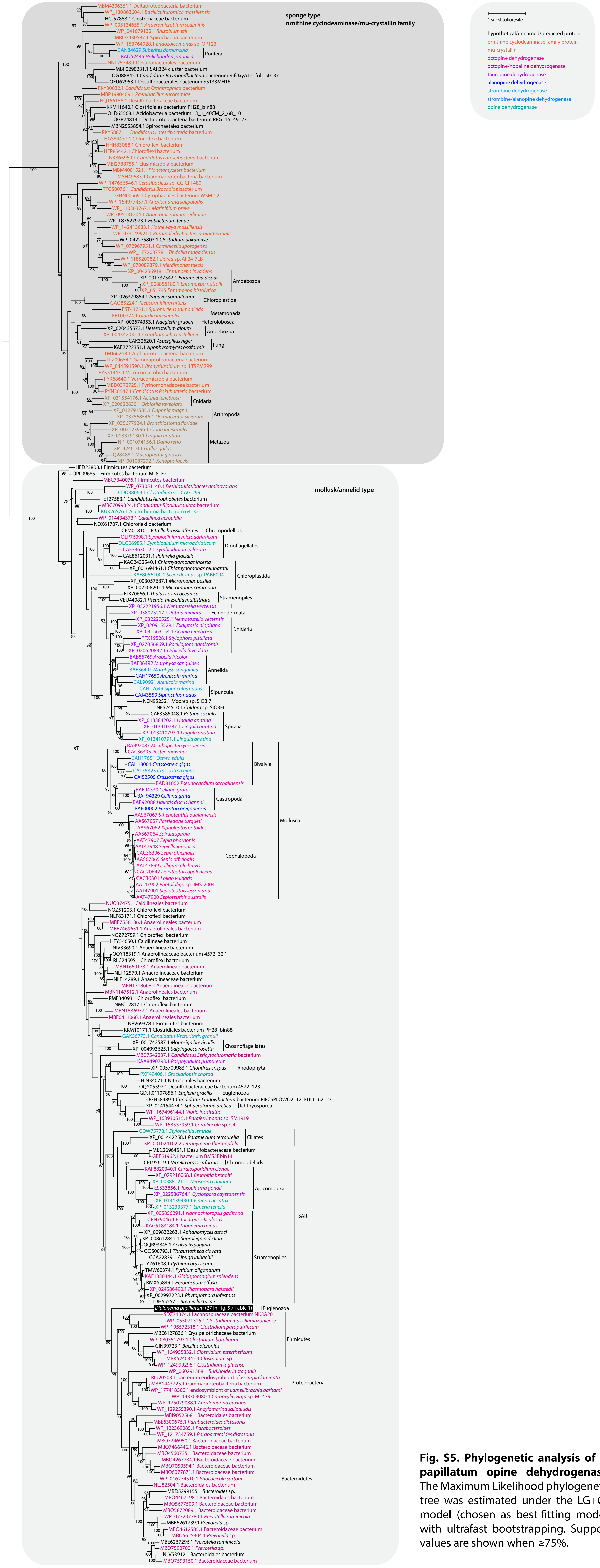

Fig. S5. Phylogenetic analysis of D. papillatum opine dehydrogenase. The Maximum Likelihood phylogenetic tree was estimated under the LG+G4 model (chosen as best-fitting model) with ultrafast bootstrapping. Support values are shown when ≥75%.
