# Supplementary material for: Highly flexible metabolism of the marine euglenozoan protist Diplonema papillatum
Source: BMC Biol. 2021 Nov 24;19:251. doi: 10.1186/s12915-021-01186-y (PMC8611851; doi:10.1186/s12915-021-01186-y)
Supplement: Supplementary file 2 — Additional file 2: File S1. Original gel of Coomassie-stained SDS-PAGE. Lanes 2-5 correspond to protein profiles shown in Fig. 1D. File S2. Original autoradiograms. Lanes 3-4 of 14C-labeled proteins (a), monosaccharides (b), lipids (c), and fatty acids (d) are shown in Additional file 1: Fig. S3A. File S3. Original western blots. Lanes 6-9, 1-4, 2-5, and 2-5 of immunodetected phosphoglucose isomerase (a), enolase (b), α-tubulin (c), and succinate dehydrogenase subunit I (d), respectively, are shown in Fig. 5. [file 12915_2021_1186_MOESM2_ESM.pdf]

Highly flexible metabolism of the marine euglenozoan protist *Diplonema papillatum*

Ingrid Škodová-Sveráková[1,2,#,*], Kristína Záhonová[1,3,#], Valéria Juricová[1,4], Maksym Danchenko[5], Martin Moos[6], Peter Baráth[5,7], Galina Prokopchuk[1], Anzhelika Butenko[1,8], Veronika Lukáčová[7], Lenka Kohútová[5], Barbora Bučková[2], Aleš Horák[1,4], Drahomíra Faktorová[1,4], Anton Horváth[2], Petr Šimek[6], and Julius Lukeš[1,4,*]

[1] Institute of Parasitology, Biology Centre, Czech Academy of Sciences, České Budějovice (Budweis), Czech Republic

[2] Faculty of Natural Sciences, Comenius University, Bratislava, Slovakia

[3] Faculty of Science, Charles University, BIOCEV, Vestec, Czech Republic

[4] Faculty of Sciences, University of South Bohemia, České Budějovice (Budweis), Czech Republic

[5] Institute of Chemistry, Slovak Academy of Sciences, Bratislava, Slovakia

[6] Institute of Entomology, Biology Centre, Czech Academy of Sciences, České Budějovice (Budweis), Czech Republic

[7] Medirex Group Academy, n.o., Trnava, Slovakia

[8] Faculty of Science, University of Ostrava, Ostrava, Czech Republic

[#] These authors contributed equally to this work

[*]Corresponding authors: jula@paru.cas.cz; skodovaister@gmail.com

Additional file 2: Files S1-S3

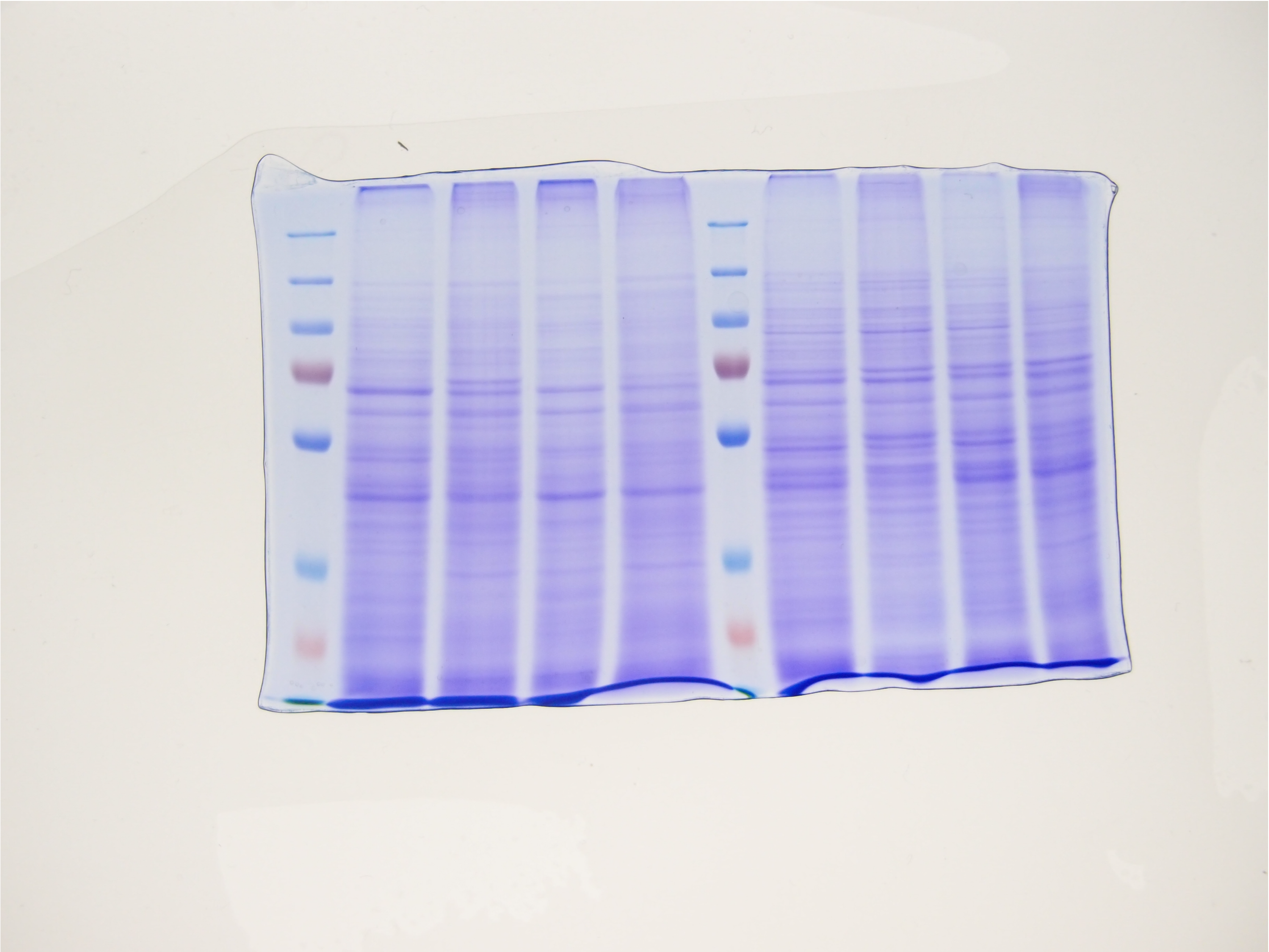

File S1. Original gel of Coomassie-stained SDS-PAGE. Lanes 2-5 correspond to protein profiles shown in Fig. 1D.

a)

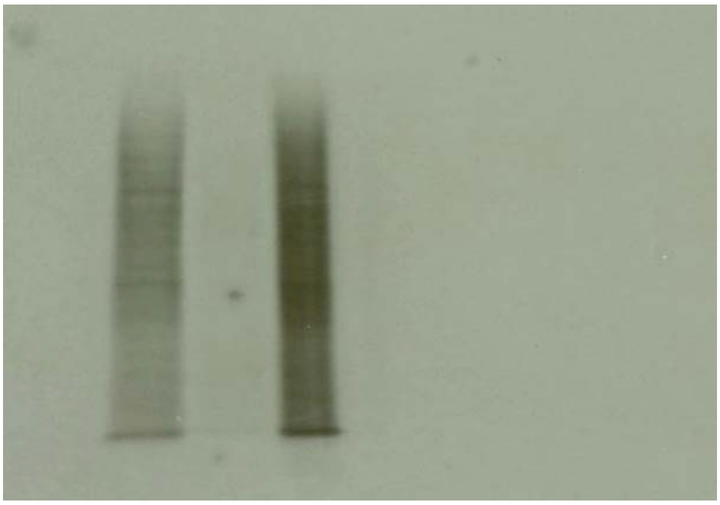

b)

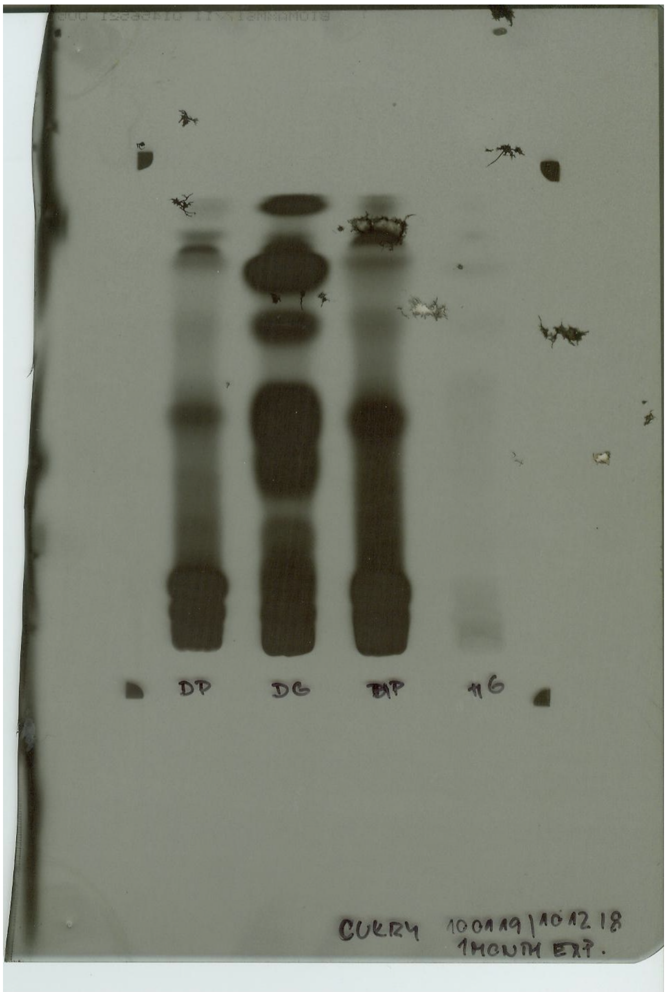

c)

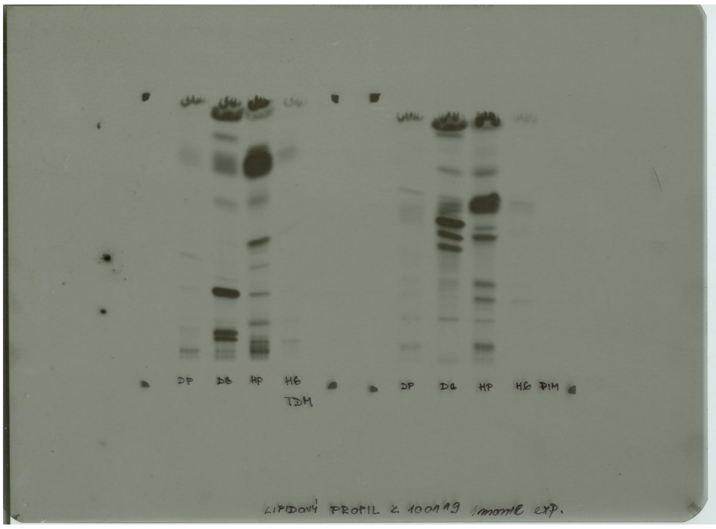

d)

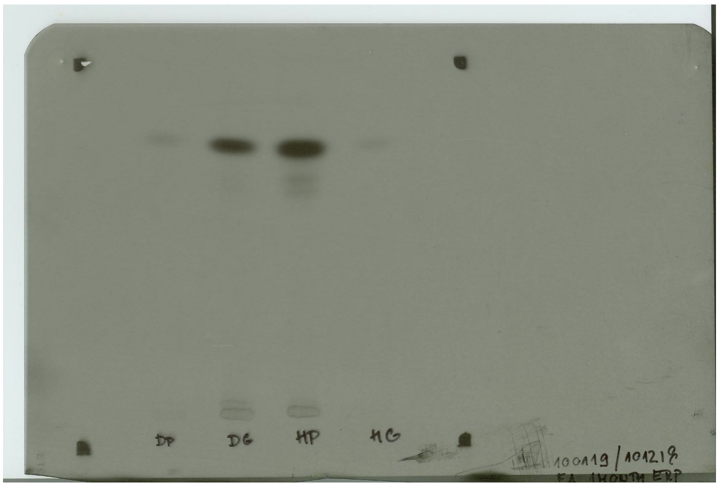

File S2. Original autoradiograms. Lanes 3-4 of ^{14}C-labelled proteins (**a**), monosaccharides (**b**), lipids (**c**), and fatty acids (**d**) are shown in Additional file 1: Fig. S3A.

a)

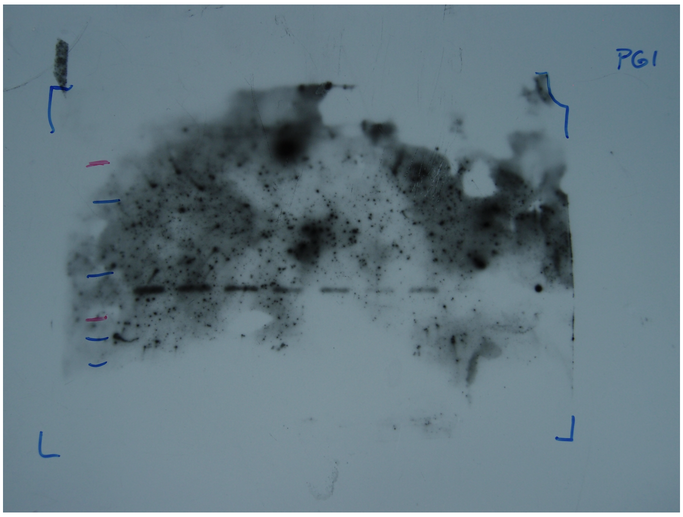

b)

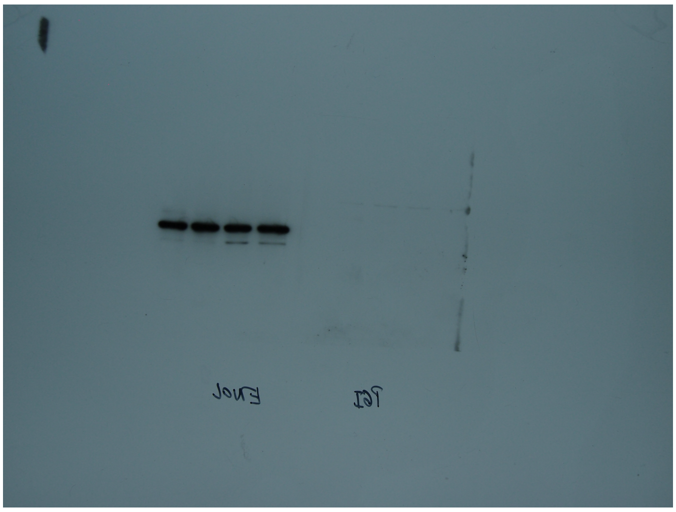

c)

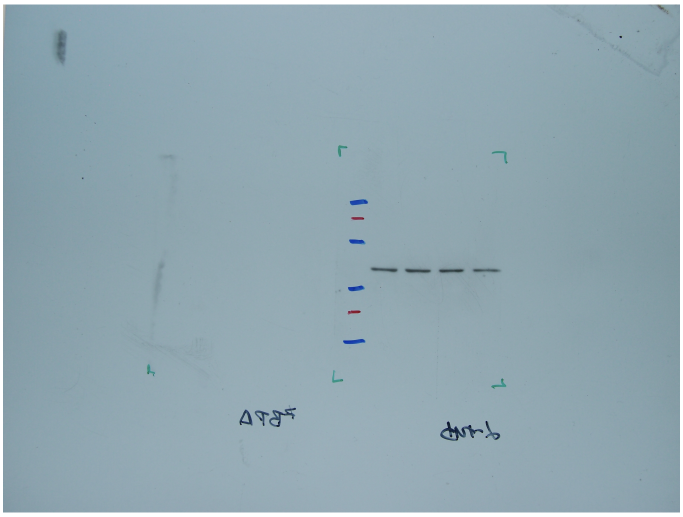

d)

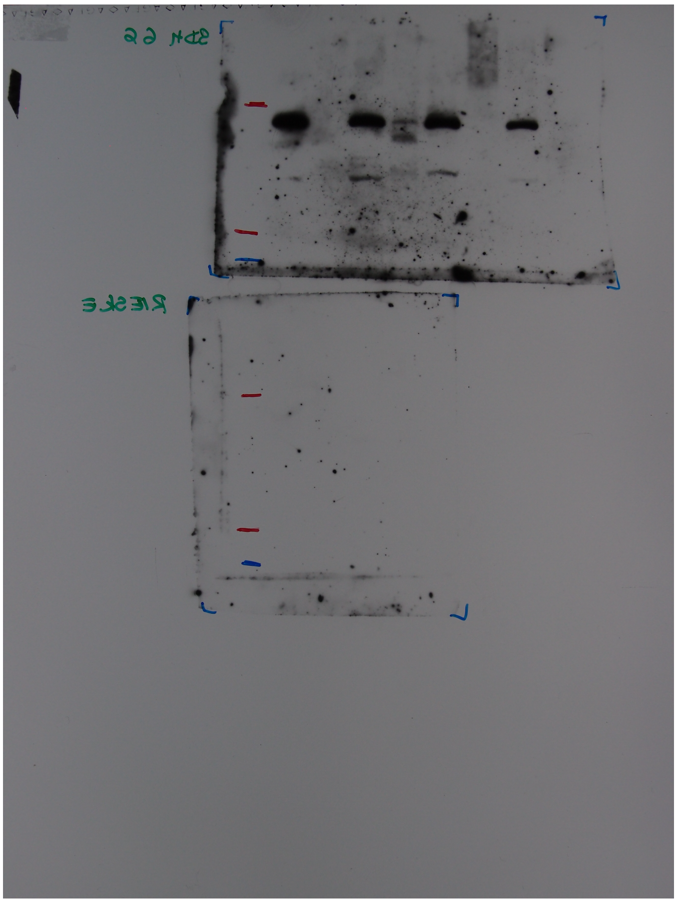

File S3. Original western blots. Lanes 6-9, 1-4, 2-5, and 2-5 of immunodetected phosphoglucose isomerase (**a**), enolase (**b**), α-tubulin (**c**), and succinate dehydrogenase subunit I (**d**), respectively, are shown in Fig. 5.
