# Supplementary material for: Highly flexible metabolism of the marine euglenozoan protist Diplonema papillatum
Source: BMC Biol. 2021 Nov 24;19:251. doi: 10.1186/s12915-021-01186-y (PMC8611851; doi:10.1186/s12915-021-01186-y)
Supplement: Supplementary file 5 — Additional file 5: Data S1. KEGG maps. [file 12915_2021_1186_MOESM5_ESM.zip › ko00010.html]

keggsvg


 
