# Supplementary material for: Highly flexible metabolism of the marine euglenozoan protist Diplonema papillatum
Source: BMC Biol. 2021 Nov 24;19:251. doi: 10.1186/s12915-021-01186-y (PMC8611851; doi:10.1186/s12915-021-01186-y)
Supplement: Supplementary file 6 — Additional file 6: Data S1. Identification and annotation of the uncommon metabolites of D. papillatum by LC-HRMS analysis. [file 12915_2021_1186_MOESM6_ESM.pdf]

Highly flexible metabolism of the marine euglenozoan protist *Diplonema papillatum*

Ingrid Škodová-Sveráková[1,2,#,*], Kristína Záhonová[1,3,#], Valéria Juricová[1,4], Maksym Danchenko[5], Martin Moos[6], Peter Baráth[5,7], Galina Prokopchuk[1], Anzhelika Butenko[1,8], Veronika Lukáčová[7], Lenka Kohútová[5], Barbora Bučková[2], Aleš Horák[1,4], Drahomíra Faktorová[1,4], Anton Horváth[2], Petr Šimek[6], and Julius Lukeš[1,4,*]

[1] Institute of Parasitology, Biology Centre, Czech Academy of Sciences, České Budějovice (Budweis), Czech Republic

[2] Faculty of Natural Sciences, Comenius University, Bratislava, Slovakia

[3] Faculty of Science, Charles University, BIOCEV, Vestec, Czech Republic

[4] Faculty of Sciences, University of South Bohemia, České Budějovice (Budweis), Czech Republic

[5] Institute of Chemistry, Slovak Academy of Sciences, Bratislava, Slovakia

[6] Institute of Entomology, Biology Centre, Czech Academy of Sciences, České Budějovice (Budweis), Czech Republic

[7] Medirex Group Academy, n.o., Trnava, Slovakia

[8] Faculty of Science, University of Ostrava, Ostrava, Czech Republic

[#] These authors contributed equally to this work

[*]Corresponding authors: jula@paru.cas.cz; skodovaister@gmail.com

Additional file 6

Data S1. Identification and annotation of the uncommon metabolites of *Diplonema papillatum* by LC-HRMS analysis.

A) Gonyol, posESI HRMS, CID MS/MS spectrum, $[M+H]^+$ = 179.07.

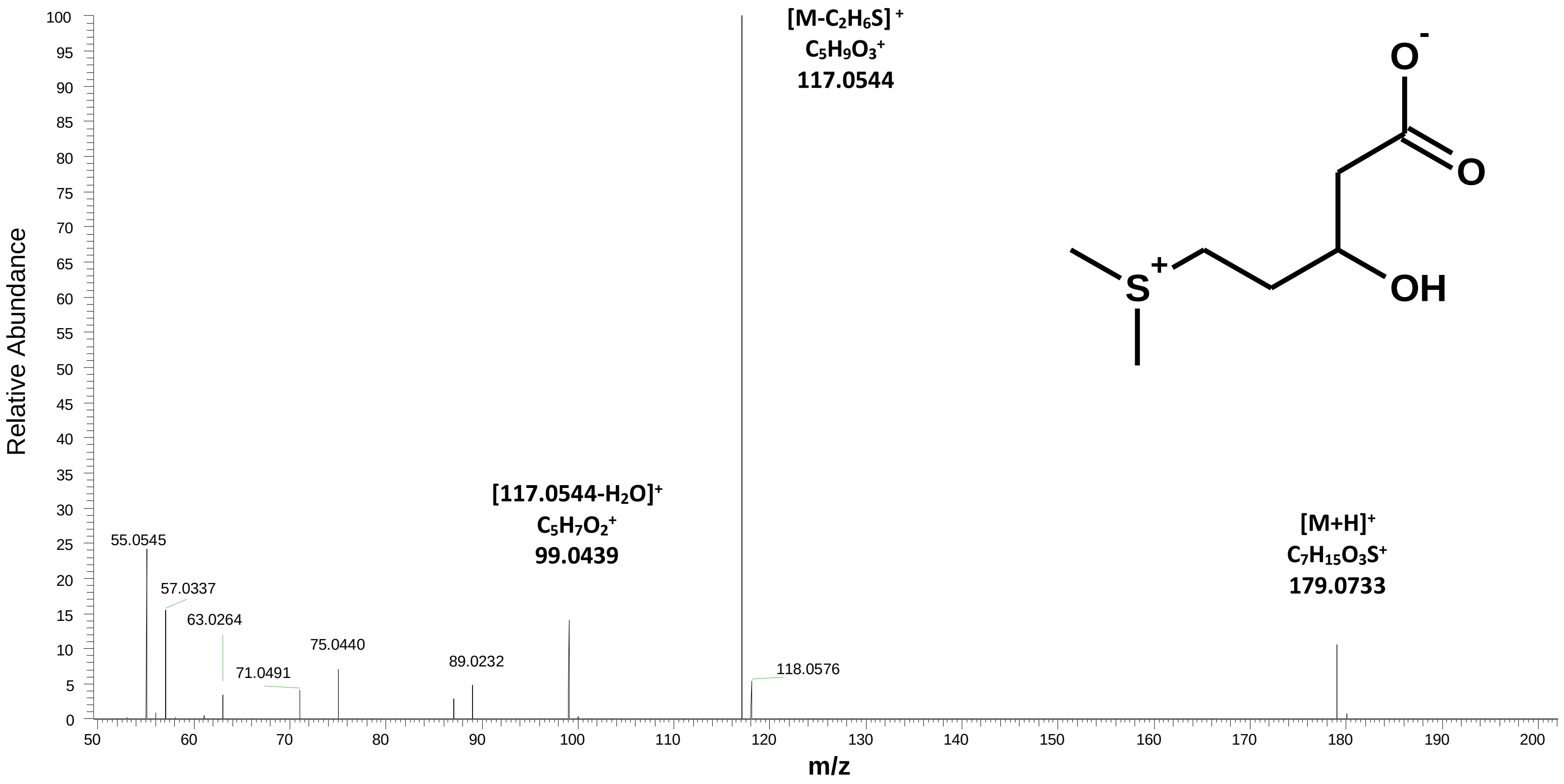

The recorded MS/MS data correspond to the Gonyol data reported in reference [SI1-R1].

B) DMSP, posESI CID MS/MS spectrum, $[M+H]^+$ = 135.05.

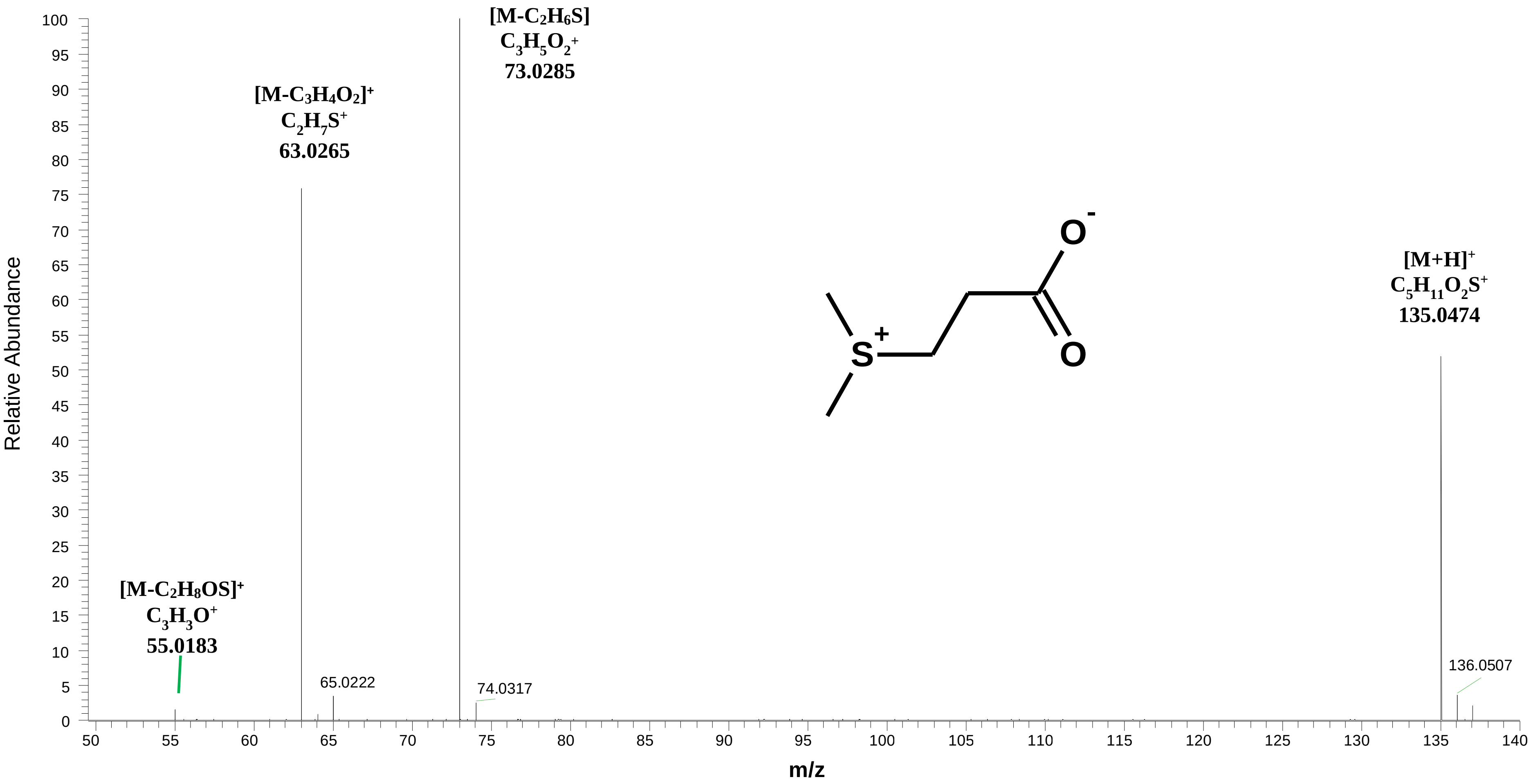

The MS/MS spectrum is in accordance with the data presented in reference [SI1-R2].

C) Dimethylseleniopropionate (DMSeP), a background subtracted posESI HRMS spectrum showing **$[M+H]^+$ = 182.9919.** All most abundant selenium isotopes raniging from ^{76}Se to ^{82}Se were detected. (**a**) A real posESI HRMS spectrum of the P- sample showing all major stable selenium isotopes of DMSeP. (**b**) A theoretical isotopic envelope of the metabolite having the $C_5H_{10}O_2Se$ elemental composition.

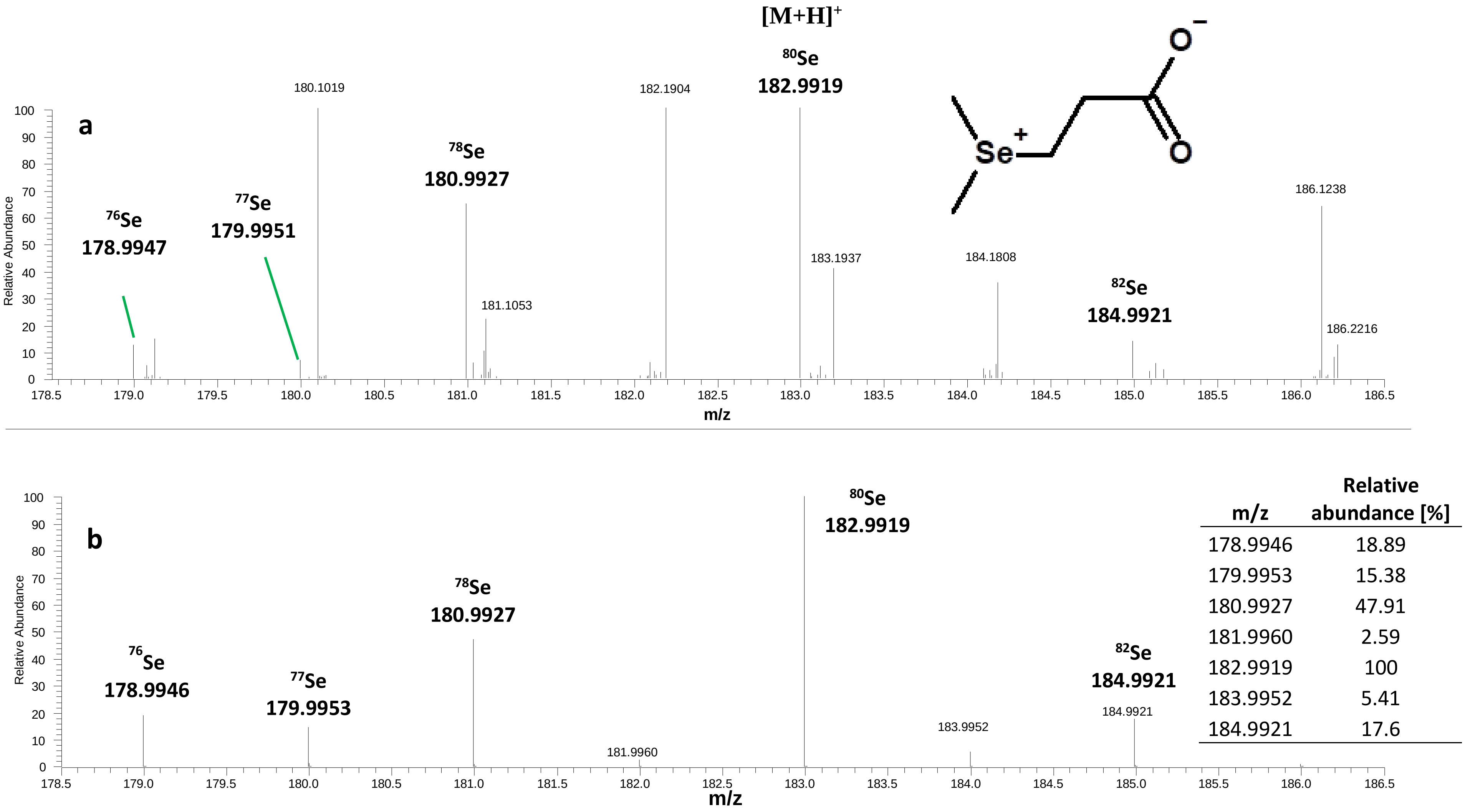

m/z	Relative abundance [%]
178.9946	18.89
179.9953	15.38
180.9927	47.91
181.9960	2.59
182.9919	100
183.9952	5.41
184.9921	17.6

D) Glyceromethyl-3-alanine betaine (GMAB, UIPAC name: 3-(2,3-dihydroxypropoxy)-2-[(trimethylammonio)methyl]propanoate), posESI HRMS, CID MS/MS spectrum, $[M+H]^+$ = 236.15 →

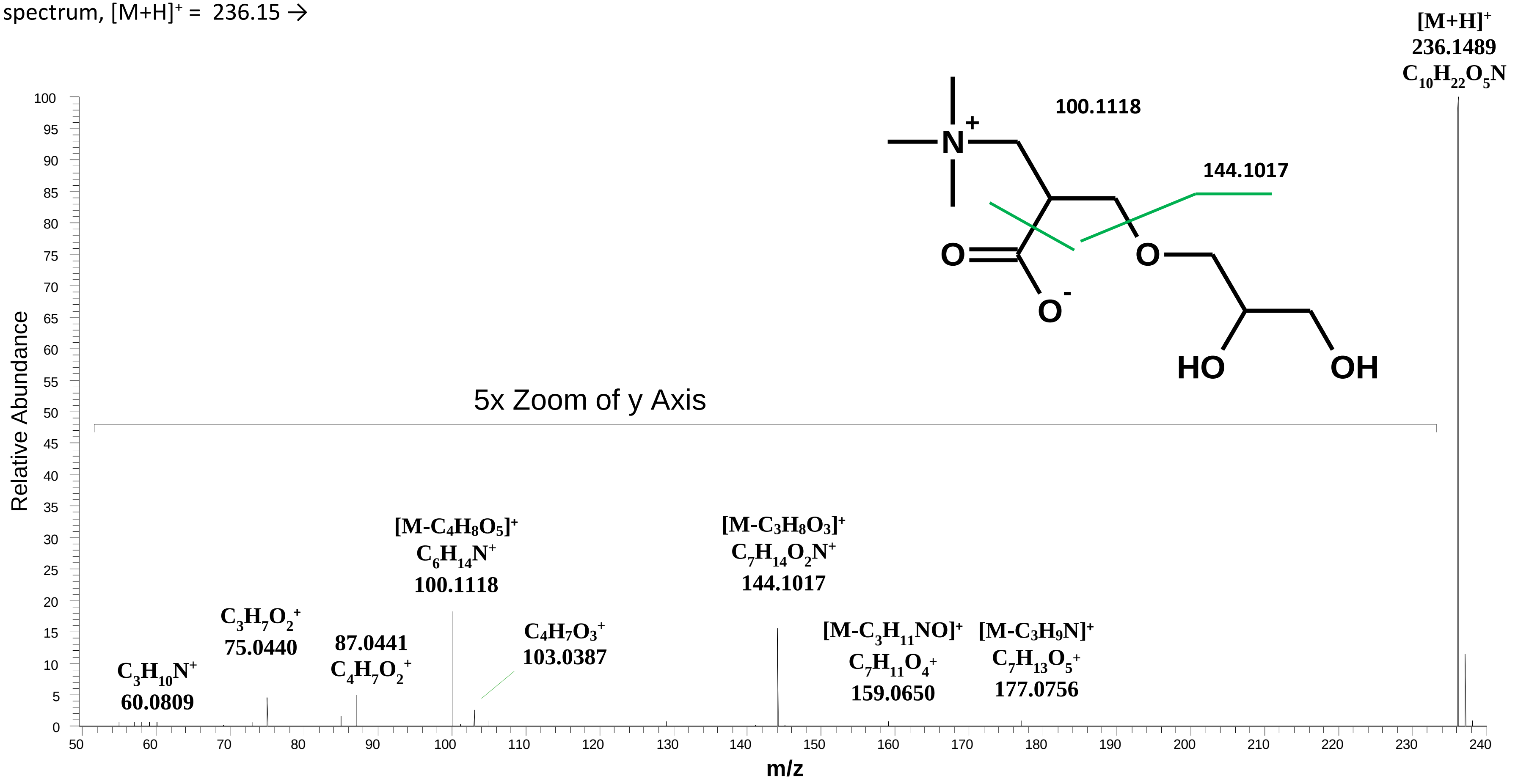

The permanent positive charge of the trimethylammonium cation directs the GMAB structure fragmentation to a glycerol loss (m/z 144.1017; $-C_3H_8O_3$) and decarboxylation providing a diagnostic m/z $C_6H_{14}N^+$ fragment (m/z 100.1118, $-CO_2$, i.e. - 43.9899). A second fragmentation route is initialized by the trimethylamine loss (m/z 177.0756; $-C_3H_9N$) followed by consecutive double water loss (m/z 159.0659, 141.0544) and finally providing fragments from the residual skeleton.

E) Glycerohomoserine betaine (GHSB), posESI HRMS, CID MS/MS spectrum, $[M+H]^+$ = 236.15 →

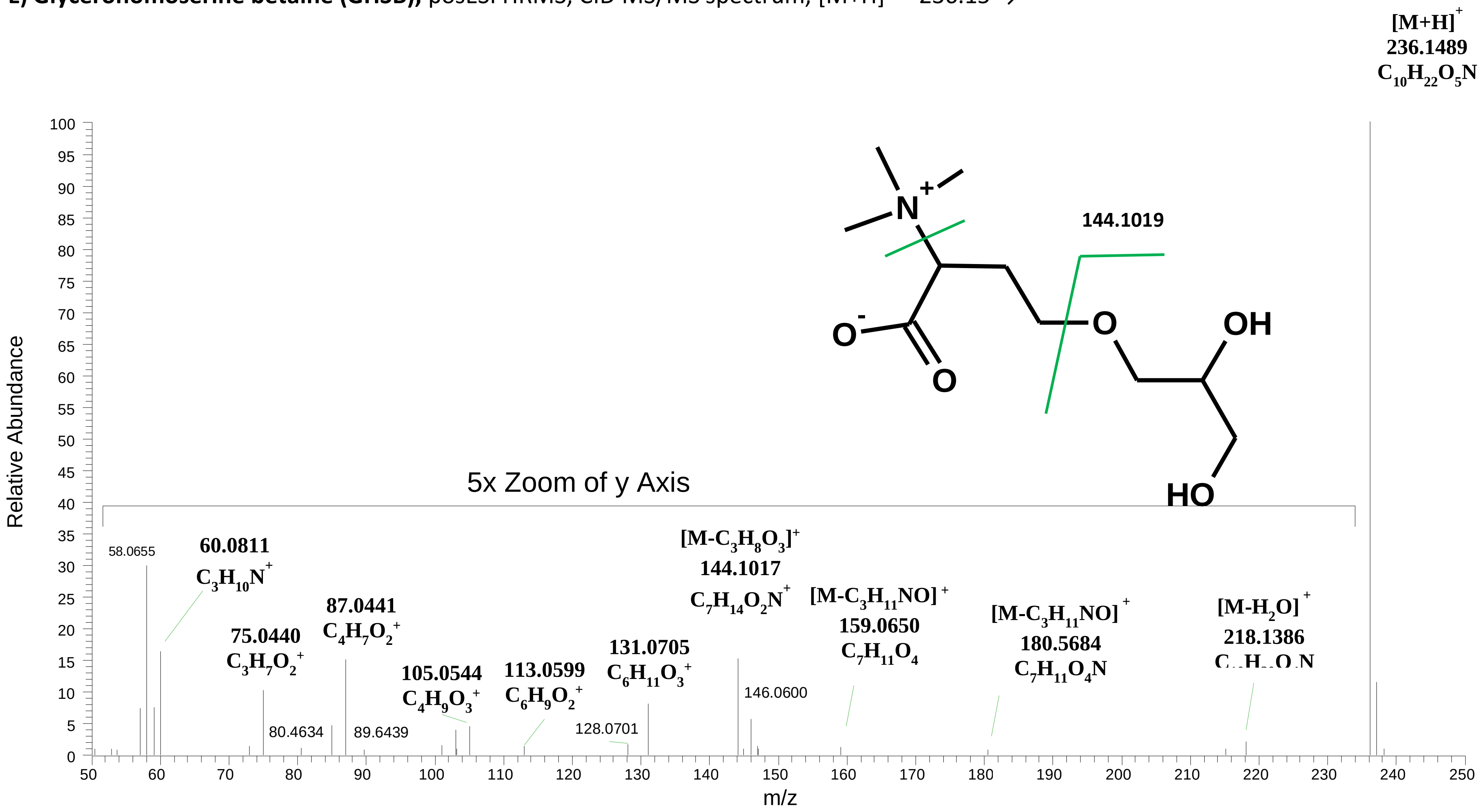

A minor detected triglycero-osmolyte **GHSB** is an isomer of **GMAB.** Its MS/MS spectrum provides again m/z 144.1019 (a glycerol loss), but the main fragmentation route occurs through the losses of trimethylamine (-59, C_3H_9N) and water(m/z 159.0656, -H_2O,) followed by a carbonyl loss (m/z 131.0705, -CO,) retaining the charge on the glycerol backbone. The characteristic trimethylammonium ion $C_3H_{10}N^+$ is also detected.

F) 3-Alanopine, posESI HRMS, CID MS/MS spectrum, $[M+H]^+$ = 162.08 →

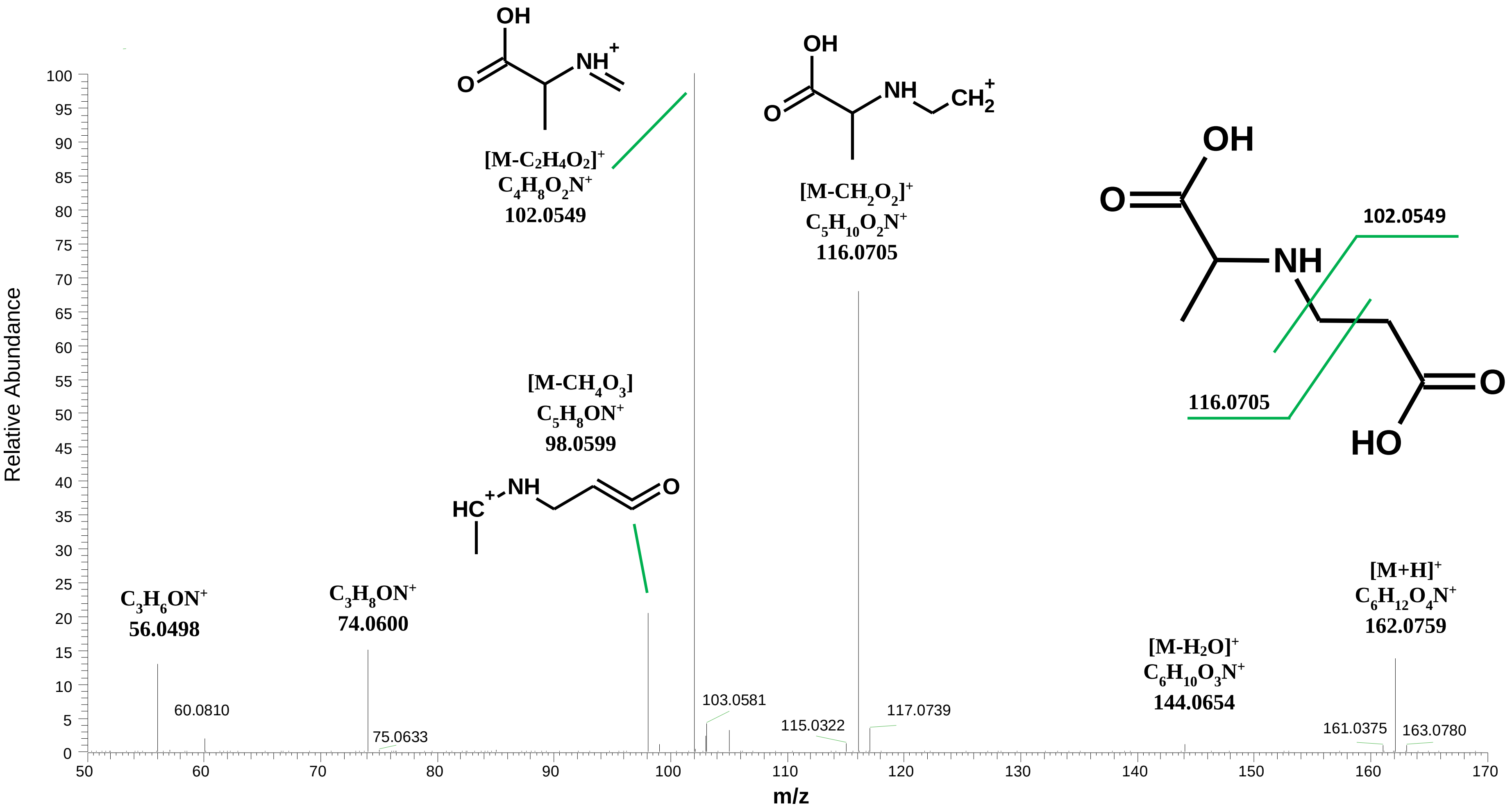

The CID MS/MS ESI spectrum was searched in open MS/MS spectral libraries, where only mass spectra of isomeric structures of 2-alanopine [Mass Bank, SI1-R2], [SI1-R3]; N-methylglutamate (Metlin ID 63297) and 2-aminoadipate (ID 4271) were found [SI1-R4]. All possible alternative isomers provided different MS/MS spectra. The 3-alanopine ESI MS/MS spectrum provides additionally a distinct m/z 102.0549 fragment. As the chemical standard was not accessible, the 3-alanopine HRMS spectrum was consulted with the Mass Frontier 7.0 fragmentation toolbox (Thermo-Fisher Scientific, USA), which confirmed the preferred formation of this fragment in the proposed structure.

G) Taurine, negESI HRMS, CID MS/MS spectrum $[M-H]^-$ = 124.01 →

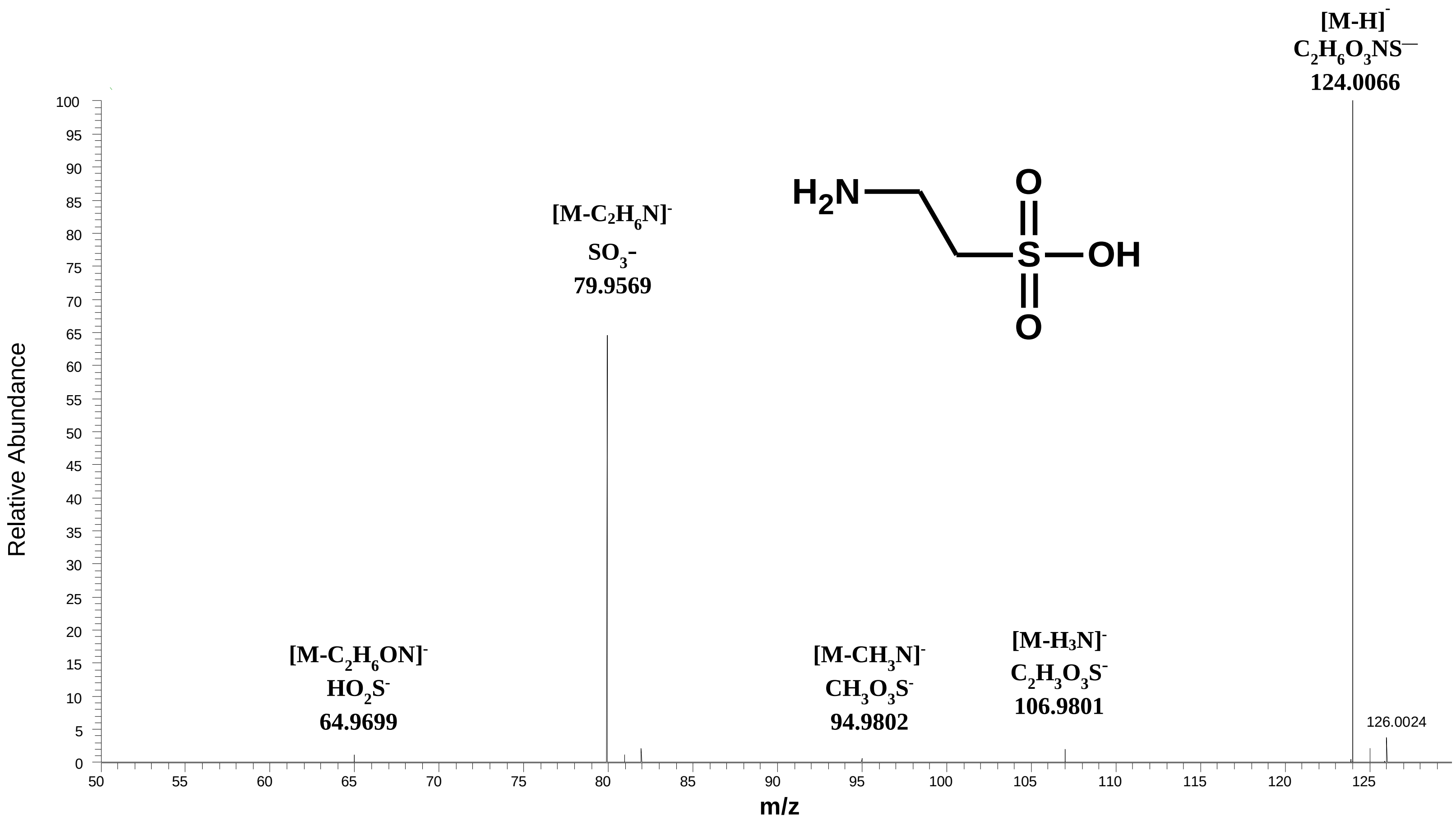

H) Tauropine, negESI HRMS, CID MS/MS spectrum, $[M-H]^-$ = 196.03 →

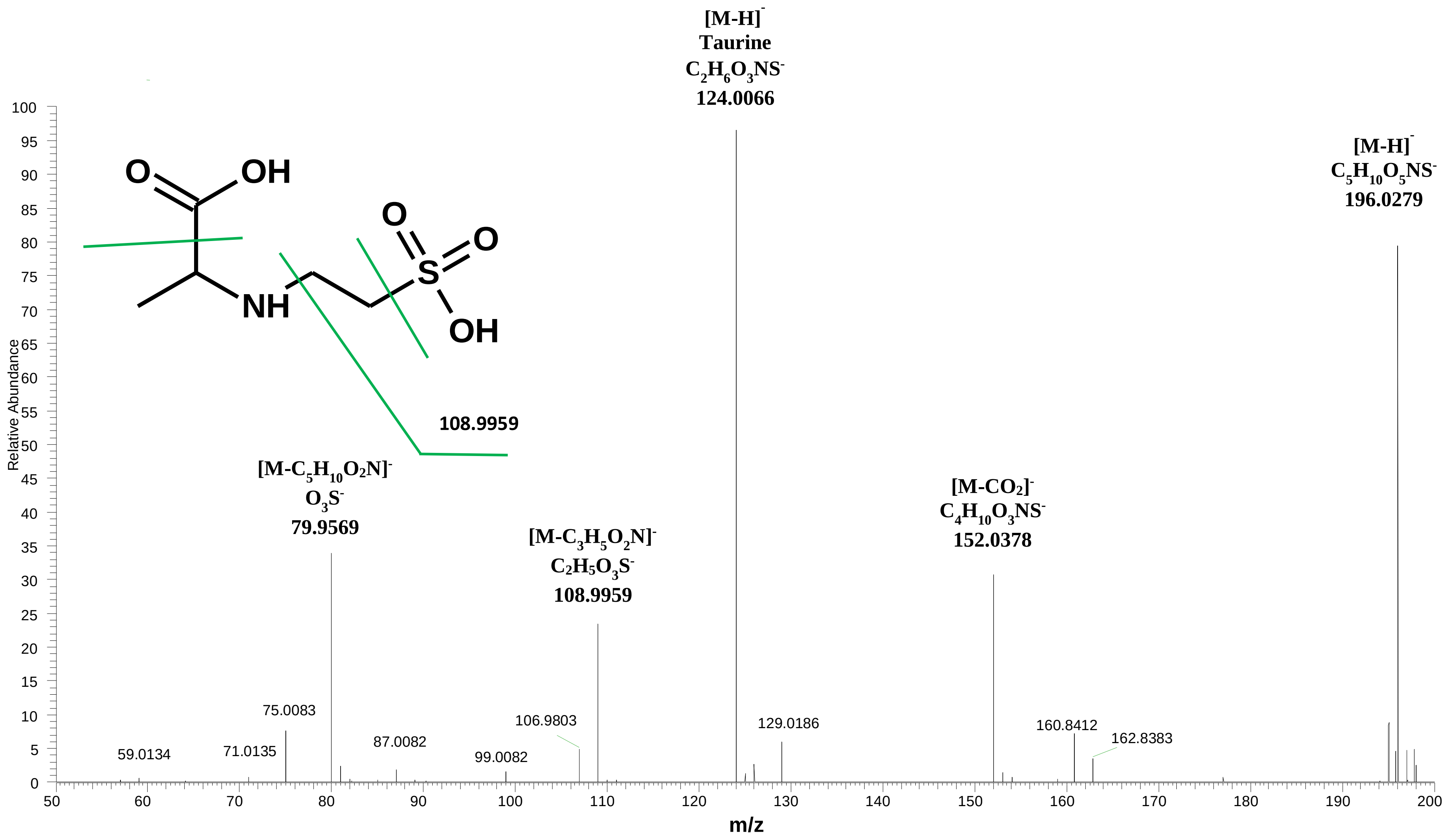

I) MGTA (C16:0, 0:0), monopalmitoylglyceromethyl-3-alanine betaine, posESI HRMS, CID MS/MS spectrum, $[M+H]^+$= 474.38 →

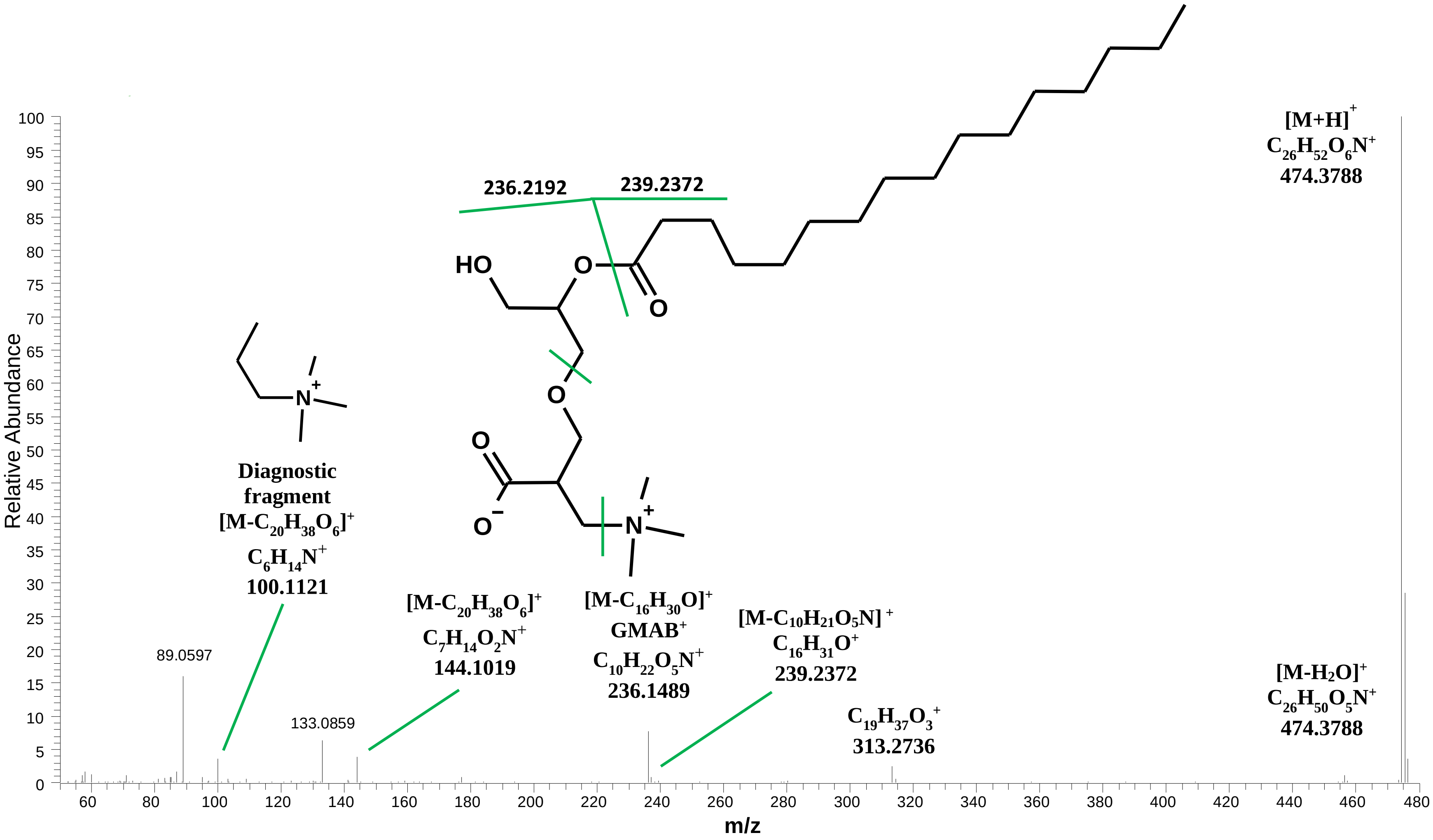

The MS/MS spectrum of the **MGTA (C16:0, 0:0)** in a pooled P+/P- sample. The GMAB diagnostic ions at the lower end of the spectrum, in particular m/z 100.1121 and 144.1019 prove the structure of the N,N,N-trimethylalanine headgroup. A loss of the hexadecyl acylium ion (m/z 239.24) show the C16:0 moiety in the monoacyl-betaine lipid structure.

J) LysoPC (16:0, 0:0), monopalmitoylglycerophosphocholine, posESI HRMS, CID MS/MS spectrum, $[M+H]^+$= 496.34 →

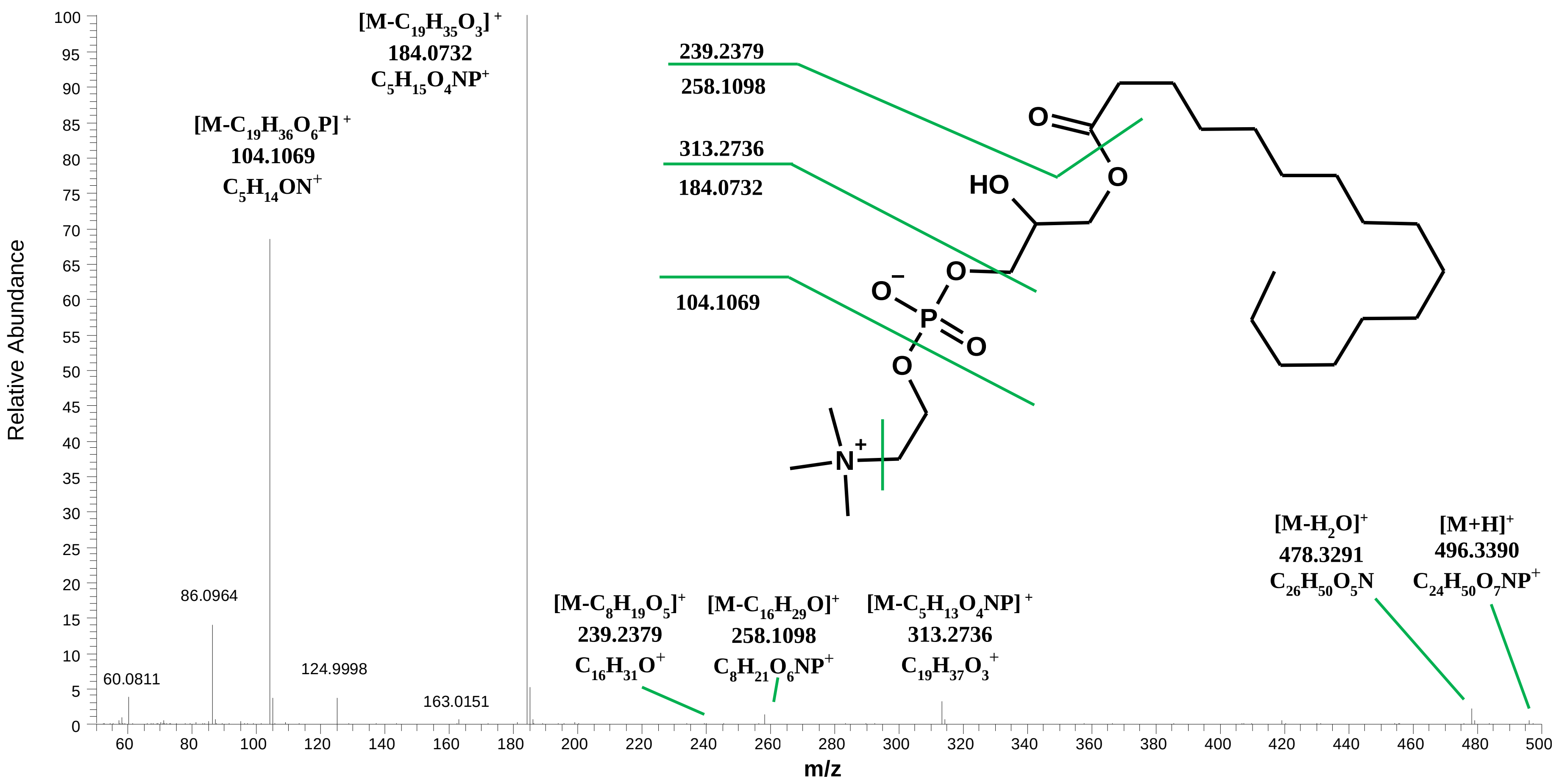

The analogous **LysoPC (16:0, 0:0)** MS/MS spectrum in a pooled R^+/R^- sample**.** The phosphocholine fragment ions dominate, the acyl moiety shows similar features as those observed in the MGTA (16:0, 0:0) MS/MS spectrum (I). For reference, e.g., refer to Metlin database, ID 182.

K) DGTA (16:0, 20:5), palmitoyl-eicosapentaenoyl-glyceromethyl-3-alanine betaine, posESI HRMS, MS/MS spectrum, $[M+H]^+$ = 758.59 →

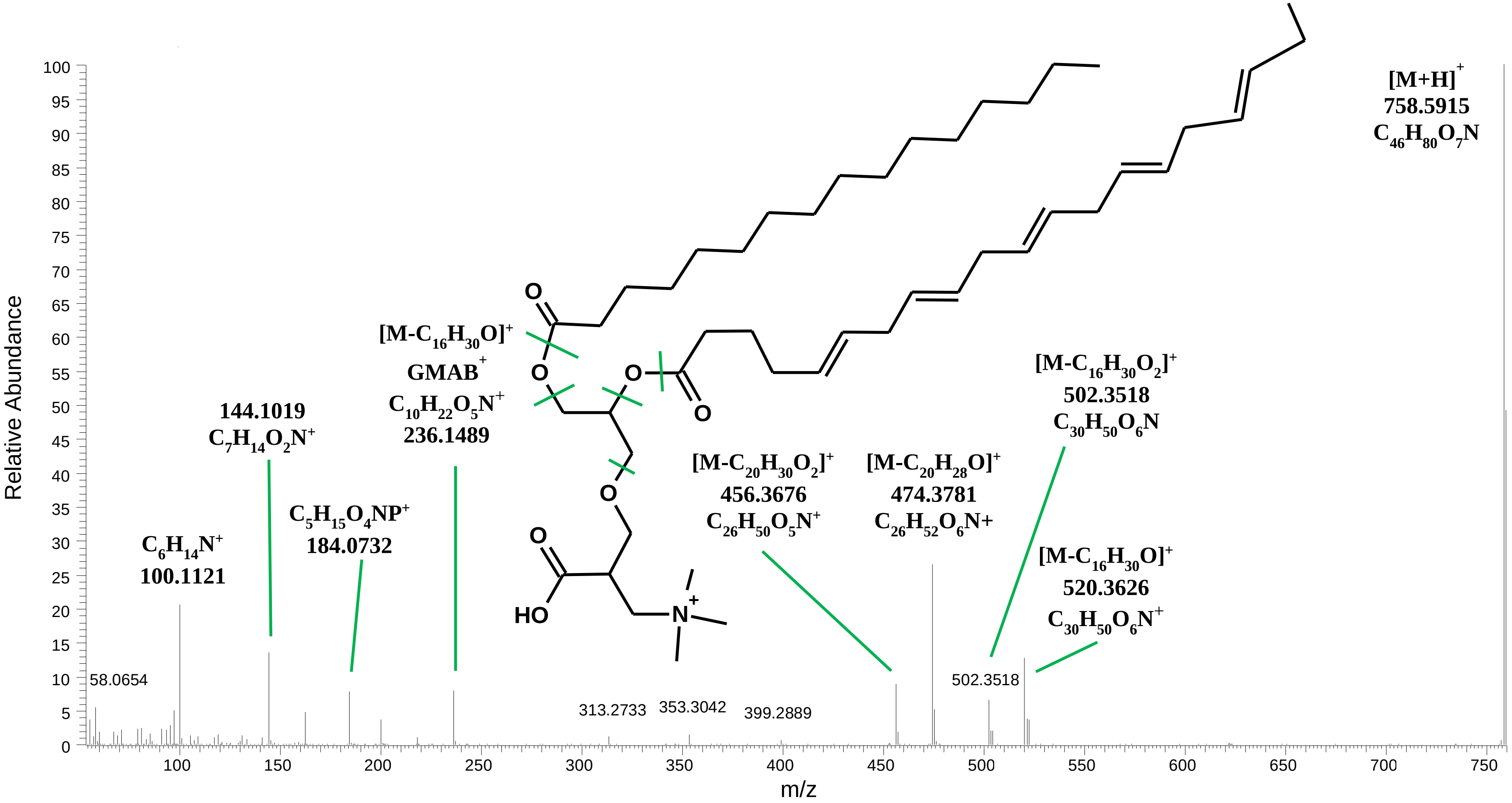

The MS/MS spectrum of the **DGTA (C16:0, 20:5),** which was highly abundant in a pooled R+/R- sample. The present GMAB ions at the lower end of the spectrum prove the betaine lipid GMAB structure. Typical acyl diagnostic ions m/z 502.3518 (a hexadecyl ketene group loss, - 238.2291, i.e. a $C_{16}H_{30}O$ loss) and m/z 520.3626 (-hexadecanoic acid, -256.397, a $C_{16}H_{32}O_2$ loss) indicate the presence of C16:0 acyl in the lipid. Similarly, the presence of the C20:5 acyl confirms the fragments m/z 456.3676 (-302.2339, a $C_{20}H_{30}O_2$ loss) and m/z 474.3781 (-284.2135, a $C_{20}H_{28}O$ loss).

L) DGTS-d9 (16:0, 16:0), 1,2-dipalmitoylglyceryl-3-*O*-4'-(*N,N,N*-trimethyl)-homoserine, posESI HRMS, CID MS/MS spectrum, $[M+H]^+$ = 721.66 →

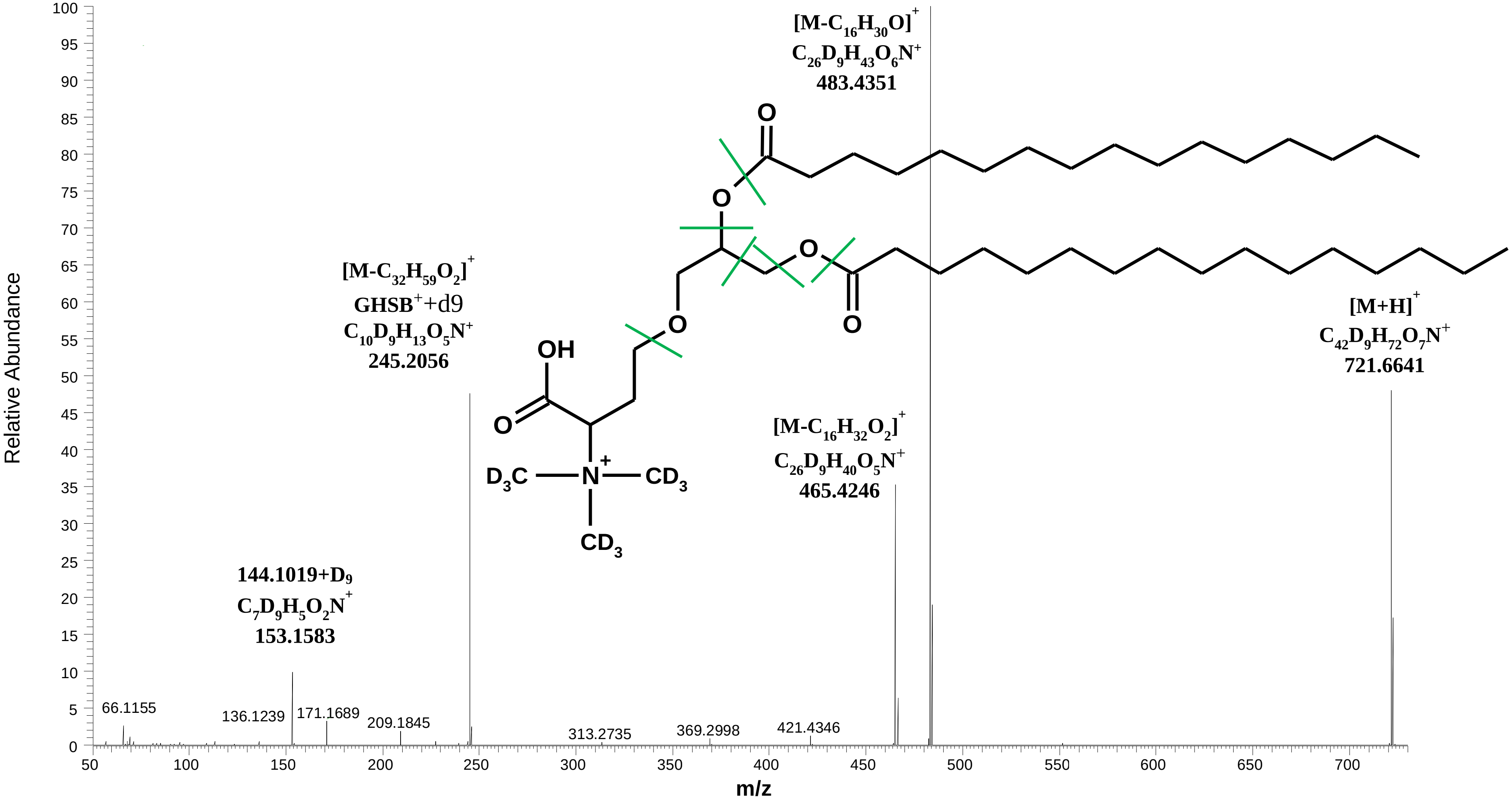

The MS/MS spectrum of the reference **DGTS-d9 (16:0, 16:0)** standard showing the characteristic +d9 mass shifts (masses 144+9, 236+9) for the GSHB headgroup, refer also to E). The absence of the distinct m/z C_6H_8N+ (m/z 101.1121), compare with D), further proves the massive production of MGTA and DGTA by *D. papillatum*. Typical acyl derived diagnostic ions m/z 483.4351 (a hexadecyl ketene group loss,- 238.2291, i.e. a $C_{16}H_{30}O$ loss) and m/z 465.4246 (- hexadecanoic acid, -256.397, a $C_{16}H_{32}O_2$ loss) prove the presence of C16:0 acyls in the nonadeuterium labeled betaine lipid.

M) PC (20:5, 22:5), eicosapentaenoyl-docosapentaenoyl-glycerophosphocholine, posESI HRMS, CID MS/MS spectrum, MH+ = 854.57→

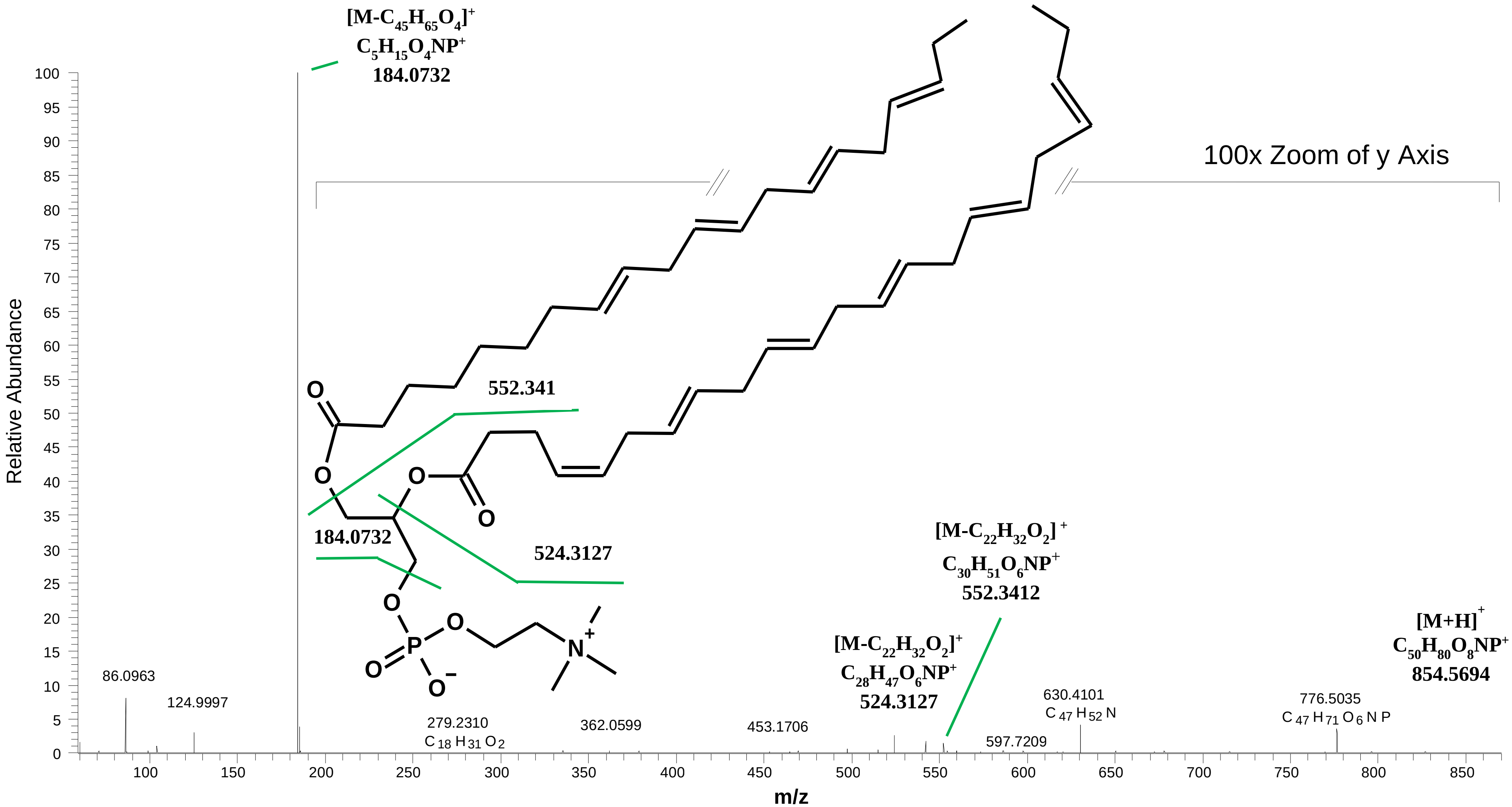

The MS/MS spectrum of the largest detected **PC (C20:5, 22:5)** highly abundant in a pooled R+/R- sample. The detected very weak fragment ions m/z 524.3127 and 552.3412 indicate a respective loss of the C20:5 (-$C_{22}H_{32}O_2$) and C22:5 ($C_{20}H_{30}O_2$) acids, which corresponds well to the largest detected circulated free fatty acids, refer to Additional file 4: Table S2.
